# An All-in-One Nanoheater and Optical Thermometer Fabricated from Fractal Nanoparticle Assemblies

**DOI:** 10.1021/acsnano.4c16452

**Published:** 2025-04-04

**Authors:** William H. Skinner, Renata L. Sala, Kamil Sokolowski, Ioana Blein-Dezayes, Natalie S. Potter, Sara Mosca, Benjamin Gardner, Jeremy J. Baumberg, Pavel Matousek, Oren A. Scherman, Nick Stone

**Affiliations:** † Department of Physics and Astronomy, University of Exeter, Exeter EX4 4QL, U.K.; ‡ Melville Laboratory for Polymer Synthesis, Yusuf Hamied Department of Chemistry, University of Cambridge, Lensfield Road, Cambridge CB2 1EW, U.K.; § Central Laser Facility, Research Complex at Harwell, STFC Rutherford Appleton Laboratory, Oxford OX11 0QX, U.K.; ∥ Nanophotonics Centre, Cavendish Laboratory, University of Cambridge, Cambridge CB3 0HE, U.K.

**Keywords:** surface-enhanced Raman scattering (SERS), Raman thermometry, thermoplasmonics, photothermal therapy (PTT), anti-Stokes, nanoassembly

## Abstract

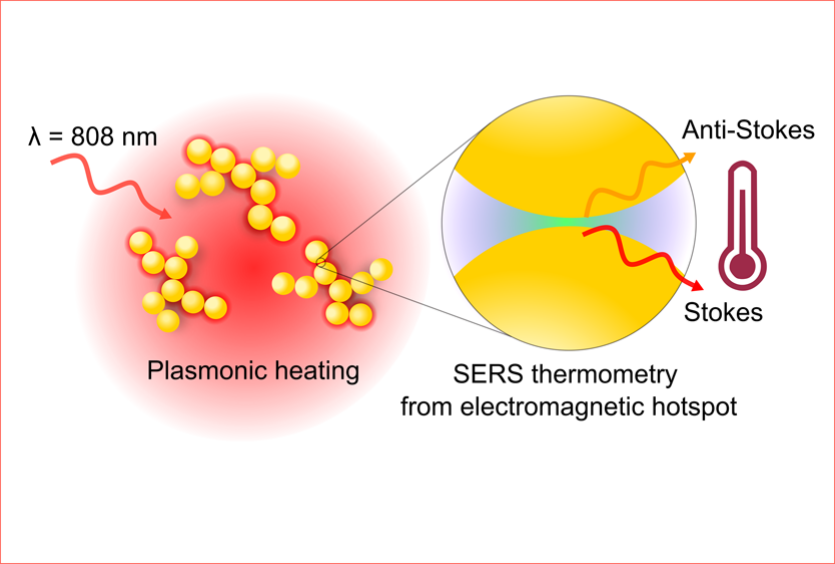

We designed and optimized a dual-functional photothermal agent that performs as a nanoheater and real-time optical thermometer by leveraging gold nanoparticle (AuNP) self-assembly and anti-Stokes thermometry. We engineered colloidally stable fractal AuNP clusters with well-defined nanogaps to absorb strongly in the near-infrared and enhance anti-Stokes vibrational modes via surface-enhanced Raman scattering (SERS) for electromagnetic (EM) hotspot-localized thermometry during plasmonic heating. Photothermal characterization and simulations of a range of AuNP building block sizes demonstrated that 40 nm AuNPs are optimum for combined plasmonic heating and SERS due to the high probability of in resonance chains within assemblies. We explored the relationship between the far-field of our AuNP clusters and the near-field enhancement of anti-Stokes modes in the context of SERS thermometry, setting out design considerations for applying SERS thermometry. Finally, using a single near-infrared (NIR) laser source, we demonstrated plasmonic heating of a colloidal system with simultaneous accurate temperature measurement from EM hotspots via the thermal information encoded in the anti-Stokes mode of surface-bound Raman reporter molecules. Ultimately, our approach could enable real-time noninvasive temperature feedback from plasmonic nanoparticles within tumor tissue environments to guide safe and effective temperature increases during cancer photothermal therapy.

## Introduction

Nanoparticle-mediated photothermal therapy (PTT) combines the light-absorbing properties of plasmonic nanoparticles with the deep tissue penetration of near-infrared light to ablate solid tumors via localized heating.^1−3^ Local temperature increase determines the efficacy of the treatment: too low, and cancer cell death does not occur; too high and off-target healthy tissue could be damaged. Techniques to monitor nanoparticle temperature and prevent collateral damage during PTT inside tissue are currently lacking. Magnetic resonance (MR) thermometry has insufficient spatial resolution, while the insertion of thermocouples into tissue is invasive.^4^ We postulate that rationally designed plasmonic nanostructures could perform as both a heat source and surface-enhanced Raman scattering (SERS) nanothermometer during PTT using a single NIR laser light source.

Since the advent of nanoparticle-mediated PTT, researchers have reported many nanoparticles as the field seeks to improve heating efficiency and increase functionality.^5−11^ These approaches typically rely on plasmonic particles, nanostructured metals or metal-like materials that, under resonant light, possess a collective oscillation of electrons called the localized surface plasmon resonance (LSPR). Heat is conducted from the plasmonic particles to their surroundings when the oscillating electrons of the excited plasmon dephase and couple to phonons.^12^

The large field enhancement at the surface of plasmonic particles and in the EM hotspots formed between them presents the opportunity for optical sensing in parallel to plasmonic heating. This enhancement is exploited in surface-enhanced Raman scattering (SERS), where concentrated electric fields at the nanoparticle surface can enhance the spectral fingerprint of molecules by up to 10^10^ for sensing applications.^13,14^

SERS measures chemical bond vibrational modes to produce a spectral fingerprint of surface-localized molecules. It also provides information on the relative population of the ground and the first vibrationally excited state of molecules, which encodes their temperature. Anti-Stokes photons arise exclusively from molecules in a vibrationally excited state, while Stokes photons arise from molecules mostly in the ground state (and any potential excited states, where present). For SERS spectra collected with a charged coupled device detector (CCD), the ratio of the anti-Stokes to Stokes peaks of a vibrational mode is linked to temperature through eq 1, derived from Boltzmann’s distribution.^15,16^
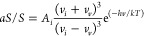
1where *aS*/*S* is the ratio of a specific vibrational mode, *A*_*i*_ is the average asymmetry factor from resonance effects and detection system spectral response effects, ν_*i*_ is the laser frequency, ν_*v*_ is the frequency of the vibrational mode, *k* is the Boltzmann constant, *h* is Plank’s constant and *T* is temperature. The commonly weak anti-Stokes signal at physiological temperatures hinders the application of anti-Stokes thermometry using spontaneous Raman scattering. However, the field enhancement of plasmonic nanoparticles can considerably enhance the Raman signal of interacting analytes, making it feasible to measure the weak anti-Stokes modes through tissue at physiological temperatures.^17^

SERS nanothermometry has been of great interest in the fundamental study of local plasmon resonance, energy transfer, and temperature at the surface of plasmonic nanoparticles during resonant excitation.^16,18−22^ However, limitations in understanding plasmon-molecule interactions and imprecise control of the near-field enhancement experienced by surface-localized analytes still present major challenges in applying SERS nanothermometry. Studies using nanoparticle aggregates to generate SERS have shown a broad distribution of *aS*/*S* ratios at single-molecule and population levels.^23,24^ This has been attributed to a combination of local heating differences among EM hotspots and variations in near-field strength. High-speed spectral characterization of single molecules in plasmonic hotspots has also demonstrated that SERS intensity fluctuations occur temporally with uneven spectral enhancements leading to anomalously intense anti-Stokes peaks.^25^

While the goal of absolute SERS temperature measurements remains challenging, we have recently shown promising evidence of measuring accurate relative temperature increases in systems where the plasmonic nanoheater and SERS nanothermometer are separate entities.^17^ Combining these functions into a single nanoparticle that generates heat and simultaneously reports local temperature would be helpful in the photothermal therapy of solid tumors, where accurate nanoparticle-localized temperature measurements are essential for effective treatment and patient safety.

In this study, we fabricate an ‘all-in-one’ SERS nanothermometer and nanoheater specifically optimized for SERS and photothermal efficiency. Using fractal gold nanoparticle clusters with precise interparticle spacing, we determine the optimum size of nanoparticle building block for fractal clusters to strongly absorb light and generate SERS in the NIR biological tissue window where light has deep penetration.^26^ We demonstrate how interparticle coupling impacts the near-field enhancement of molecules in EM hotspots in a wavelength-dependent manner and the implications of this for designing a SERS nanothermometer. Finally, we show accurate temperature measurements from the nanoparticle surface during laser exposure. These results demonstrate an ‘all-in-one’ nanoheater and SERS nanothermometer capable of simultaneously heating under NIR irradiation while providing real-time temperature measurements.

## Results and Discussion

### AuNP Cluster Fabrication

**Figure 1:**
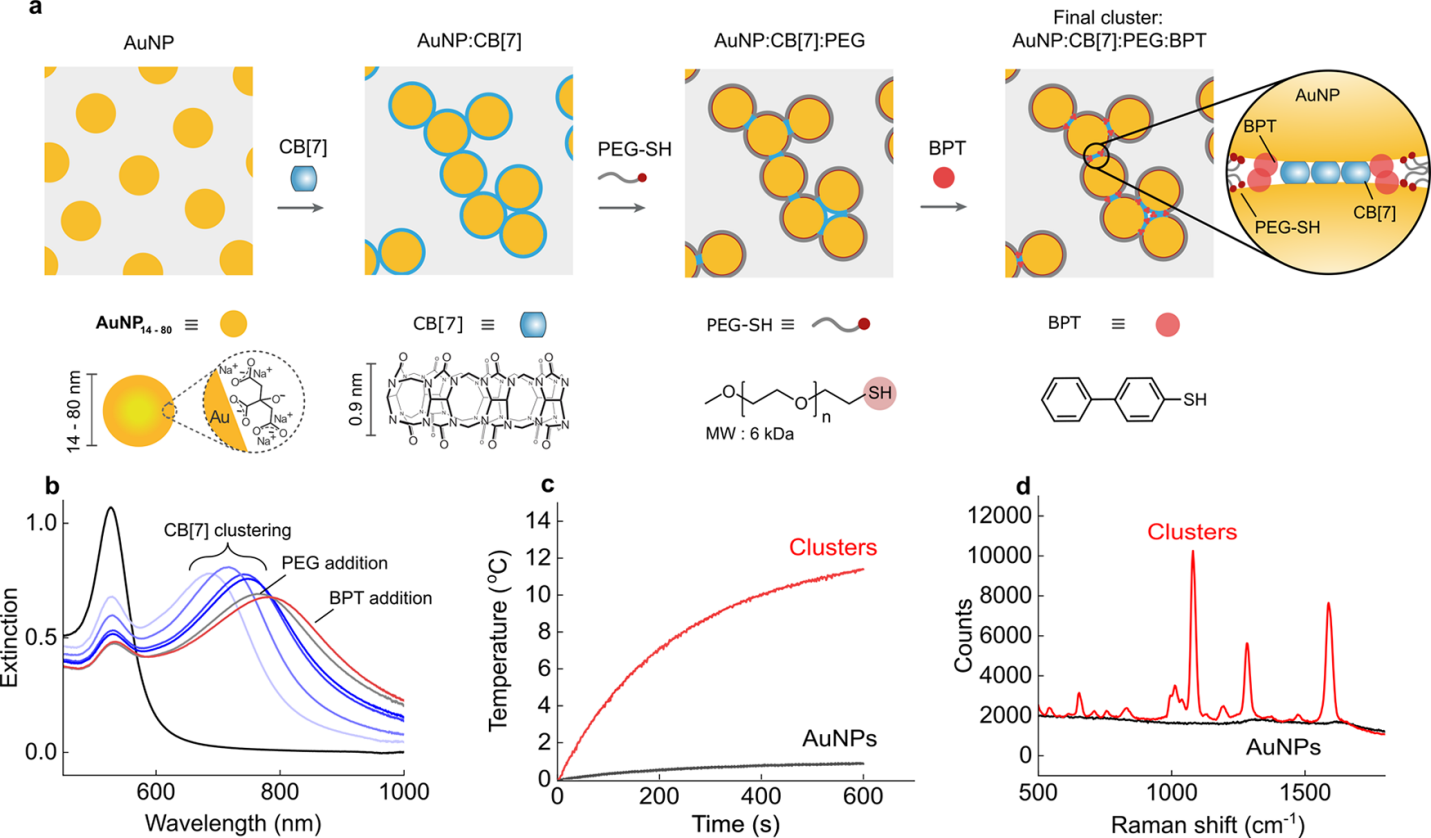
(a) Self-assembly of colloidally stable fractal AuNP clusters. AuNPs were first aggregated with CB[7] as a ‘molecular glue’ and, in a subsequent step, stabilized with PEG-SH to prevent further assembly. The resultant AuNP:CB[7]:PEG assemblies were further functionalized with SERS reporter molecule BPT. (b) Typical UV–vis spectra of AuNP colloid (*d* = 40 nm) before and after CB[7] addition. AuNPs assemble into fractal structures, and the peak plasmon mode redshifts as clusters increase in size; PEG-SH stabilization and BPT addition further redshift the plasmon mode to the NIR. (c) The temperature increase in clustered and monodisperse AuNP (*d* = 40 nm) colloid during exposure to 808 nm laser (1 W, [Au] = 0.4 mg/mL). (d) SERS spectra of clusters and BPT-functionalized AuNPs (*d* = 40 nm) at equal concentrations ([Au] = 0.4 mg/mL). The near-field enhancements in interparticle gaps generate stronger SERS relative to monodisperse AuNPs.

To achieve simultaneous plasmonic heating and SERS anti-Stokes thermometry for application in photothermal therapy, a suitable nanoconstruct is required to absorb light in the NIR and generate strong SERS to enhance the weak anti-Stokes vibrational modes. To fulfill these requirements, we first created fractal clusters of AuNPs by assembling commercially available citrate-stabilized AuNPs with cucurbit[7]uril (CB[7]) macrocycles. CB[7] is a rigid molecule with a strong binding affinity for AuNPs that, upon aggregation, creates interparticle gap spacings of 0.9 nm (Figure 1a).^27^ Plasmonic coupling along chains of CB[7]-linked AuNPs within the fractal clusters red-shifted the LSPR to the NIR region (Figure 1b), at which point cluster growth was kinetically arrested by adding poly(ethylene glycol) methyl ether thiol (PEG-SH, 6 kDa) ligands to passivate the surface and prevent further cluster growth.^28^ The addition of PEG-SH arrests the transition of monodisperse AuNP into large aggregates, creating a stabilized intermediate state of fractal aggregation. Biphenyl-4-thiol (BPT) was added to the clusters to populate interparticle gaps and act as a SERS reporter. Clusters with a significant extinction in the NIR region improved heating performance compared to monodisperse AuNPs (Figure 1c). At the same time, field confinements in the interparticle gaps generated an intense BPT SERS spectrum (Figure 1d). Field enhancement of ∼200 is predicted in CB-defined interparticle gaps of 40 nm AuNPs when the plasmon mode and laser wavelength are matched.^29^ We used BPT as our Raman reporter molecule because of its large Raman cross-section relative to CB[7], resulting in a stronger signal (Figure S2–1). The final BPT-functionalized clusters showed good biocompatibility (Figure S2–2). The facile nature of our AuNP assembly process enabled stable clusters to be formed in minutes from commercially available building blocks and applied to a range of AuNP diameters, thus facilitating rapid optimization of the AuNP parameter space for photothermal properties and SERS efficiency.

### Photothermal Properties of AuNP Clusters

The size of the AuNP building block used to create fractal clusters is a key design parameter in optimizing the light-to-heat conversion efficiency of clusters. Published works on AuNP clusters for PTT have yet to characterize this parameter fully.^30−33^

**Figure 2:**
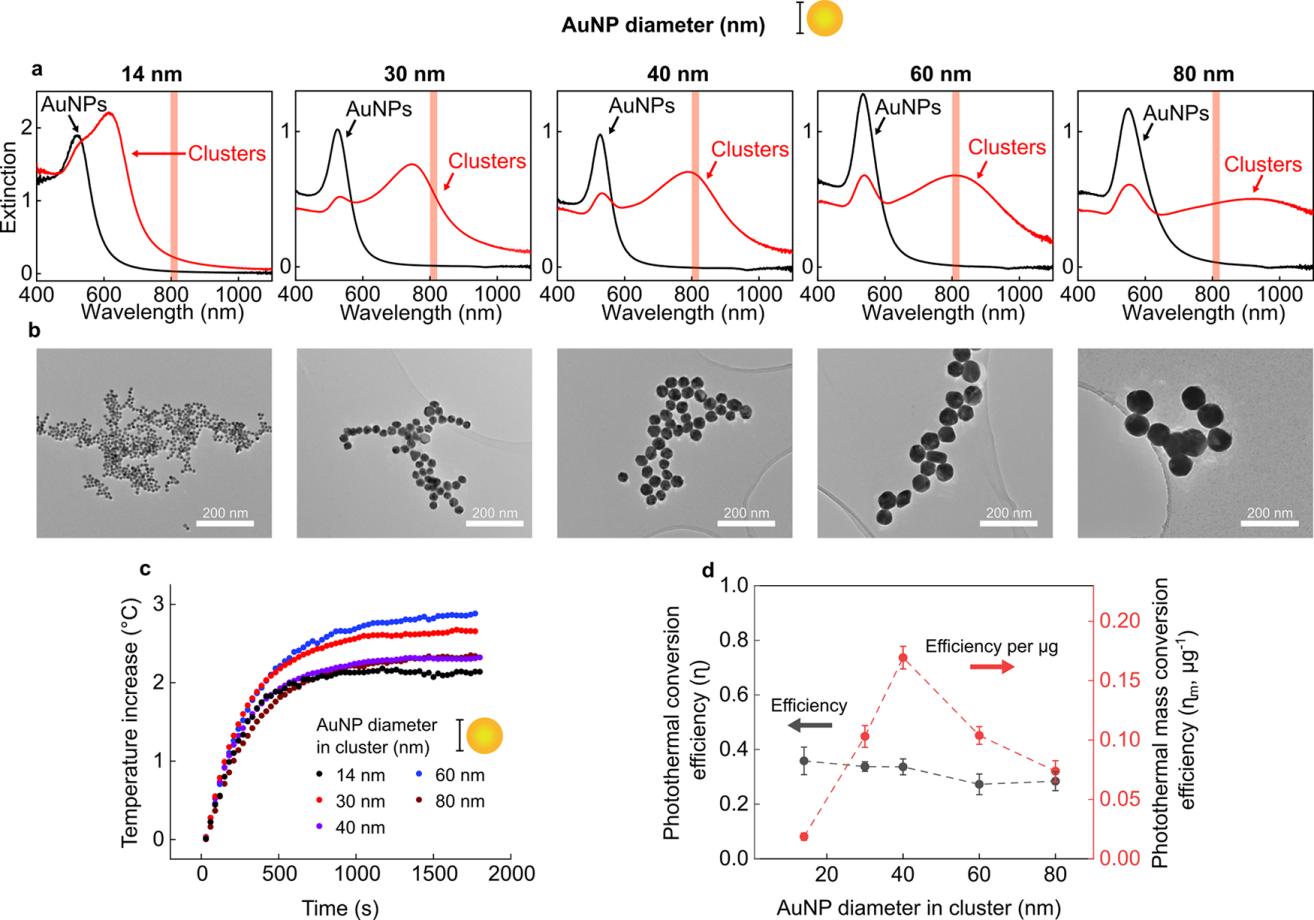
(a) UV–vis spectra of starting AuNP (14–80 nm) colloids (black) and assembled AuNP clusters (red). The shaded red line indicates the laser wavelength used for PTT (808 nm). (b) Example transmission electron microscopy (TEM) images of AuNP (14 nm–80 nm) clusters. All TEM images were taken with the same magnification. (c) Representative experimental heating data used to calculate photothermal conversion efficiency (η) (1 W, 808 nm laser). The cluster solutions were diluted to ε_808_ ≈ 0.1. (d) The photothermal conversion efficiency (η) and photothermal mass conversion efficiency (η_m_) of nanoclusters assembled from AuNPs of different diameters (*n* = 3 independent repeats).

Photothermal conversion efficiency (η) is widely used in the field to evaluate the light-to-heat conversion efficiency of plasmonic nanoparticles.^11,30,34,35^ η is the experimentally determined fraction of extinguished laser light absorbed and converted to heat by a nanoparticle colloid. Monodisperse gold nanoparticles have a well-established relationship between size and photothermal conversion efficiency. As nanoparticle size increases, the photothermal conversion efficiency decreases as a smaller proportion of light is absorbed and more is scattered.^36^ We explore the impact of AuNP building block diameter on η by assembling clusters from 5 sizes of AuNP between 14 and 80 nm (Figure 2a,b) and evaluating their photothermal conversion efficiencies (Figure 2c,d).

Solutions of each cluster were heated with an 808 nm laser, and η was calculated from the resulting heating and cooling data (Figures 2c, S2–3, and S2–4). η remained remarkably consistent for clusters fabricated from 14 to 40 nm AuNP building blocks (η ≈ 0.34) with a slight decrease for 60 and 80 nm AuNP clusters (η ≈ 0.27) (Figure 2d). Hence, independent of building block size, approximately 30% of extinguished laser light is absorbed and contributes to heating. This is similar to the efficiency measured for gold-silica nanoshells (η = 0.33), a plasmonic nanoparticle already applied to PTT in human patients.^35^

The hydrodynamic diameter of clusters tuned to the wavelengths in Figure 2 ranged from 230 to 100 nm (Figure S2–5). For monodisperse AuNP, the η measured at 532 nm (a wavelength unsuitable for PTT) follows a size dependence, decreasing from 0.8 to 0.65 between 15 and 50 nm AuNP.^36^ However, when AuNP are assembled into larger fractal clusters, and η is evaluated at 808 nm (a wavelength suitable for PTT), the increased size of AuNP clusters relative to the nanoparticle building blocks lowers η, as an increased fraction of extinguished light is scattered and less is absorbed. Previous work exploring the relationship between η and nanoparticle size has shown that highly scattering colloids, such as the clusters in this work, effectively increase the path length of the laser within the sample, increasing the probability of photon absorption and masking predicted decreases in η.^30^ Hence, it is likely that, when assembled into clusters, any minor size dependence is masked by the reabsorption of scattered photons within the solution. Therefore, η remains consistent at ∼30% for clusters of AuNP with 14–80 nm diameters.

In vivo delivery studies of metallic nanoparticles typically measure accumulation with inductively coupled plasma mass spectrometry (ICP-MS), which yields an AuNP concentration with units of μg/g of tissue.^36,37^ Hence, it is advantageous to compare the relative heating performance of nanoheaters as a response per μg of nanoparticle and, in doing so, capture both η and the extinction cross-section of the clusters in a single measurement.^37^ To achieve this, we measured the photothermal mass conversion efficiency (η_m_) (Figure S2–6), which captures the fraction of 808 nm light absorbed per μg of Au in the laser path. Calculating η_m_ revealed a relationship between the diameter of the AuNP building block and the heating achieved per μg of clusters (Figure 2d). As AuNP building block size increased from 14 to 40 nm, the η_m_ increased from 0.02 to 0.17 μg^–1^, before decreasing to 0.07 μg^–1^ for 80 nm AuNPs. These data suggest that 40 nm is the optimum AuNP building block diameter to fabricate clusters for photothermal therapy applications.

We rationalized the empirical finding that 40 nm AuNP clusters are optimum for photothermal applications by simulating the plasmonic coupling along chains of 1–10 AuNPs of 14–80 nm in diameter (Figure S2–7). The optical properties of AuNP clusters can be deconstructed into the contributions of simpler 1D AuNP chains within the clusters and previous work has shown that interparticle coupling effectively saturates above 10 AuNPs in a linear chain.^27,29,38^ These simplified chains estimate the interparticle coupling required to create AuNP clusters in resonance with the 808 nm laser light used for PTT. The plasmon mode of 14 and 30 nm AuNP chains do not redshift sufficiently, reaching 625 and 725 nm, respectively, for a chain of ten AuNPs (Figure S2–7). We show this experimentally by allowing 14 and 30 nm AuNP to cluster for extended periods of time without kinetic arrest (Figure S2–8). Even when micron-scale clusters are formed after hours of CB[7]-mediated assembly, the cluster mode is not in resonance with the 808 nm laser. Meanwhile, 60 and 80 nm chains exhibit large redshifts such that, beyond short chains of AuNPs, the plasmon mode is out of resonance with the laser, and the superposition of plasmon modes from multiple chain lengths is broad. However, chains of seven or more 40 nm AuNPs create plasmon modes with substantial laser line overlap, plateauing close to 800 nm. Therefore, fractal 40 nm AuNP clusters have the highest probability of containing multiple chains in resonance with the 808 nm light sources and, hence, most effectively couple with incoming photons to generate heat.

### Influence of Cluster Parameters on Anti-Stokes SERS

**Figure 3:**
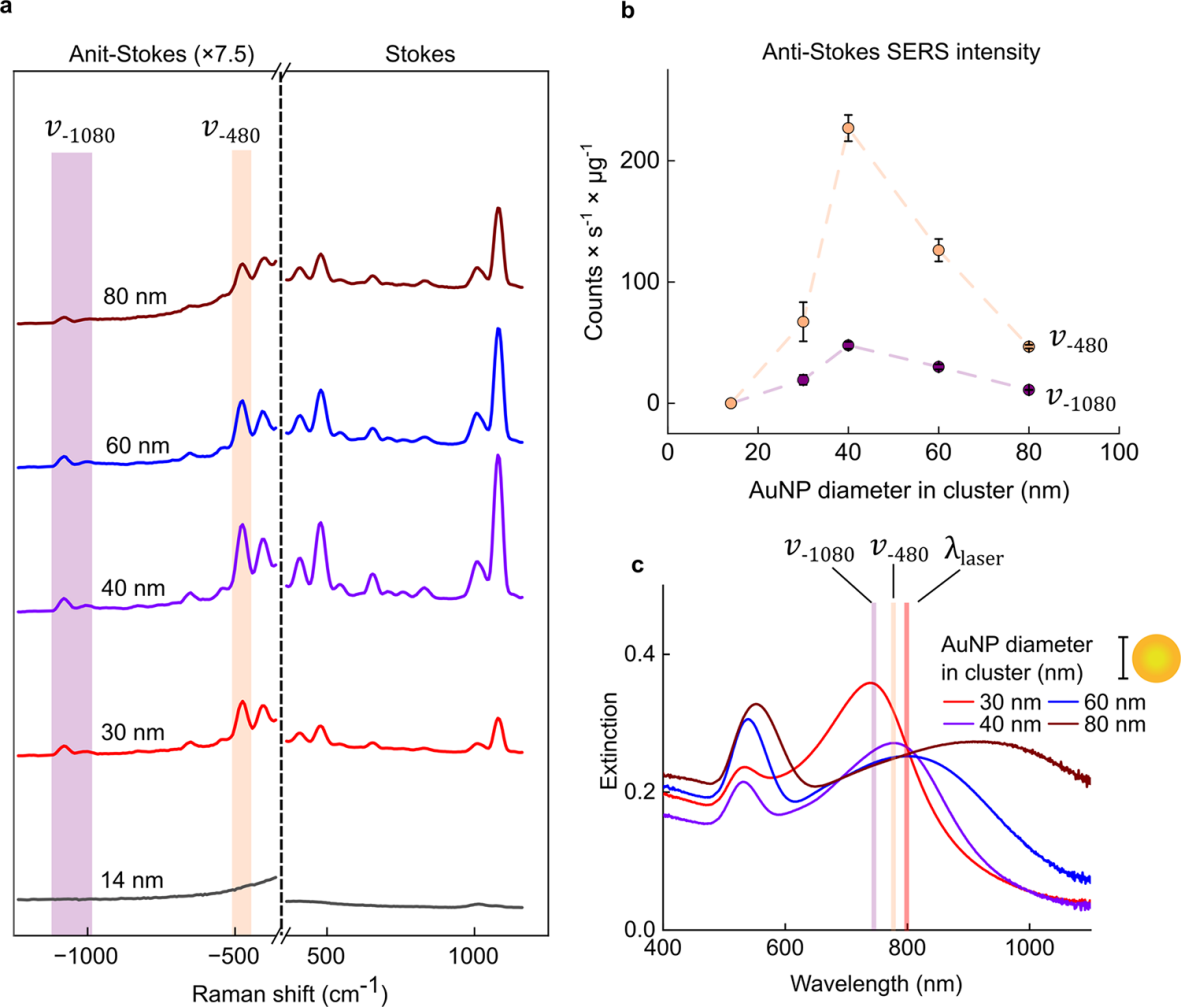
(a) SERS spectra of clusters fabricated from 14–80 nm AuNPs (ε_808_ ≈ 0.25). Zoomed-in anti-Stokes and Stokes spectra are presented in Figure S3–1. (b) Anti-Stokes SERS intensity per μg Au for ν_–480_ and ν_–1080_ modes from each cluster type. Error bars are ± SD of *n* = 3 batches of clusters. Peak intensity was calculated after baseline subtraction. (c) UV–vis spectra of clusters fabricated from different AuNP building block diameters. λ_laser_ indicates excitation wavelength, ν_–480_ and ν_–1080_ highlight the wavelength of emitted anti-Stokes photons from each vibrational mode relative to the plasmon of the clusters. The UV–vis spectrum of 14 nm AuNP clusters was not included because no anti-Stokes signal was detected.

To perform as both a nanoheater and optical thermometer, AuNP clusters must exhibit strong SERS and plasmonic heating. The limiting factor for anti-Stokes thermometry is signal intensity. The small population of molecules in a vibrationally excited state at body temperature produces a low probability of generating anti-Stokes photons. To determine the optimum AuNP clusters for SERS temperature sensing, we collected SERS spectra from each cluster type (Figures 3a and S3–1) in solutions diluted to an equal extinction at the laser wavelength (ε_808_ ≈ 0.25).

We analyzed two vibrational modes of interest from BPT for temperature sensing: 480 cm^–1^ (ν_480_) and 1080 cm^–1^ (ν_1080_), both assigned to phenyl ring modes coupled to the C–S bond (Figure 3b,c).^39,40^ The ν_480_ mode is the lowest energy peak unaffected by the notch filter and, therefore, the most intense peak in the anti-Stokes region suitable for temperature sensing. The ν_1080_ mode is much lower in intensity but, according to 1, is expected to have a larger relative increase per °C (Figure S3–2), yielding improved temperature sensitivity at 37–50 °C, the temperature window relevant for PTT.

Figure 3b,c shows that 40 nm AuNP clusters most effectively enhance the anti-Stokes signal of ν_480_ and ν_1080_. From the electromagnetic (EM) SERS theory, optimum enhancement occurs when both the incoming laser field (ω_in_) and the Raman shifted photon emitted (ω_out_) are in resonance with the plasmon mode of the metal nanoparticle (eq 2).^41,42^
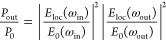
2where  is the EM enhancement factor, *E*_0_ is the incident field and *E*_loc_ is the increased local field at the nanoparticle surface at each frequency. The strongest anti-Stokes intensity for both modes was achieved with 40 nm AuNP clusters because their plasmon resonance balances both terms most effectively. The clusters are simultaneously in resonance with the 808 nm laser (ω_in_) but slightly blueshifted, so also in resonance with anti-Stokes frequencies (ω_out_). The remaining clusters miss this ‘goldilocks’ zone where both terms are balanced: 30 nm clusters are suited for anti-Stokes enhancement but are not in resonance with incoming photons, while 60 nm cluster modes are slightly red-shifted relative to the laser and much broader. For SERS thermometry, the plasmon mode should be optimized to resonate with both ω_*in*_ and anti-Stokes ω_out_ to maximize the enhancement of the weak anti-Stokes modes.

Although this selective EM enhancement benefits anti-Stokes signal strength, the wavelength-dependent nature of the enhancement presents a significant challenge for SERS thermometry. While the *aS*/*S* ratio is temperature-dependent for spontaneous Raman, in the case of SERS, it also depends on the clusters’ plasmon resonance and, therefore, the extent of interparticle coupling within nanoparticle clusters.

**Figure 4:**
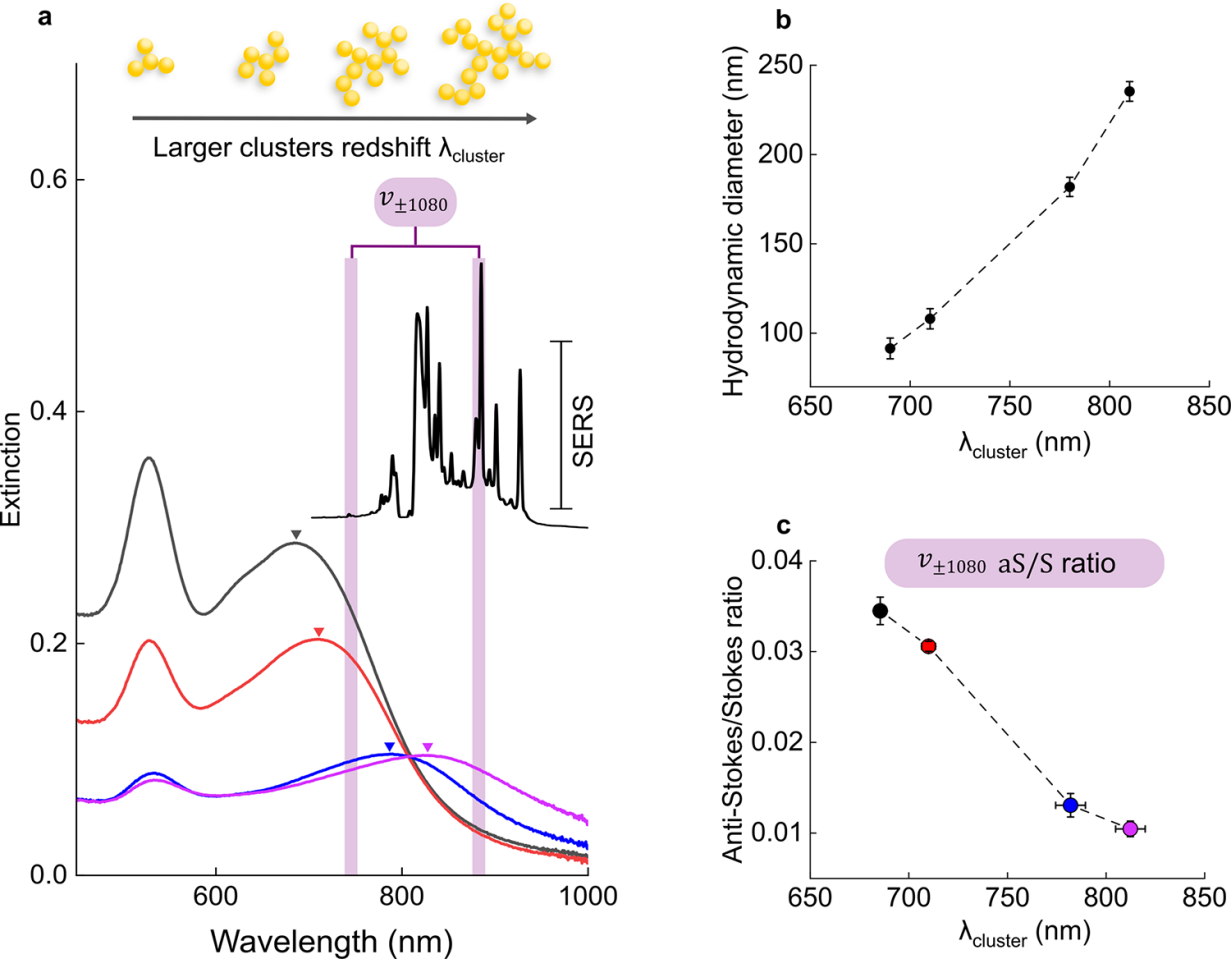
(a) UV–vis and SERS spectra of four 40 nm AuNP clusters (diluted to ε_808_ ≈ 0.1) with peak cluster plasmon wavelengths (λ_Cluster_) ranging from ∼690 to ∼810 nm (highlighted by triangles). The Stokes and anti-Stokes vibrational modes (v_±1080_) and their wavelengths are highlighted in purple. (b) Cluster hydrodynamic diameter, measured by DLS, and plotted against λ_Cluster_ (*n* = 3, technical repeats). (c) Relationship between v_1080_*aS*/*S* ratio and λ_Cluster_. Error bars are ± SD of the λ_Cluster_ and *aS*/*S* ratio for *n* = 3 batches of clusters assembled under the same conditions.

To explore the relationship between interparticle coupling, plasmon mode wavelength, and *aS*/*S* ratio, we prepared four 40 nm AuNP clusters with increasing cluster hydrodynamic diameter to create samples with peak resonance (λ_Cluster_) between ∼690 and ∼810 nm (Figure 4a,b). The *aS*/*S* ratio decreased for both ν_1080_ (Figure 4c) and ν_480_ (Figure S3–3) as cluster size increased and λ_Cluster_ red-shifted. This is driven by decreased *E*_loc_(ω_out_) resonance for anti-Stokes photons and increased resonance for Stokes scattered photons, indicating that, in our colloidal system, the far-field extinction tracks with the near-field enhancement of surface-bound molecules, consistent with previous studies on two-dimensional (2D) plasmonic substrates.^21,43^ The *aS*/*S* ratio of ν_1080_ decreased by 70% as λ_Cluster_ red-shifted from ∼690 to ∼810 nm. A 10 °C increase above body temperature is predicted to increase the *aS*/*S* ratio of ν_1080_ by only 20% (Figure S3–2). Furthermore, the influence of cluster size on plasmon mode wavelength also determines the photothermal properties of the clusters. We characterized this relationship in Figure S3–4. Increasing cluster size and redshifting the plasmon mode toward 808 nm increased the photothermal mass conversion efficiency (η_m_). Hence, the structural stability of the clusters during heating is paramount for reproducible plasmonic heating and accurate SERS temperature measurements, as changes in cluster size and interparticle coupling could affect the relative enhancement of anti-Stokes vibrational modes, leading to inaccurate SERS temperature measurements.

To test the thermal stability of 40 nm AuNP clusters, we heated them to 50 °C in a water bath for multiple cycles (Figure S3–5). We measured an initial redshift after the first heating cycle and a stable λ_Cluster_ on subsequent rounds of heating. The initial redshift was ascribed to a small irreversible increase in nanoparticle cluster size or rearrangement of BPT molecules at the nanoparticle surface following the first heating cycle. The constant λ_Cluster_ in subsequent heating cycles confirmed the clusters had suitable thermal stability to prevent *aS*/*S* ratio drift during plasmonic heating. Therefore, cluster size can be controlled to enhance anti-Stokes modes selectively, and their thermal stability ensures confidence that thermally driven changes to the vibrational excitation of surface-bound BPT molecules will be the sole influence of subsequent changes in anti-Stokes intensity.

### Combining Plasmonic Heating and SERS Thermometry

Accurate monitoring of local temperature is vital during the photothermal therapy of solid tumors; temperature elevation dictates the extent of cell death and whether cell death occurs via an inflammatory necrotic pathway or a controlled apoptotic pathway.^44,45^ Agents that act both as nanoheaters and nanothermometers could help guide therapy. We have shown that 40 nm AuNP cluster colloids absorb around ∼30% of extinguished photons (Figure 2d). The remaining photons are scattered, a fraction of these photons contribute to the SERS spectrum, potentially enabling temperature measurements from the EM hotspots between AuNPs. This presents the opportunity to use 40 nm AuNP clusters as nanoheaters and SERS nanothermometers under irradiation from a single laser source.

**Figure 5:**
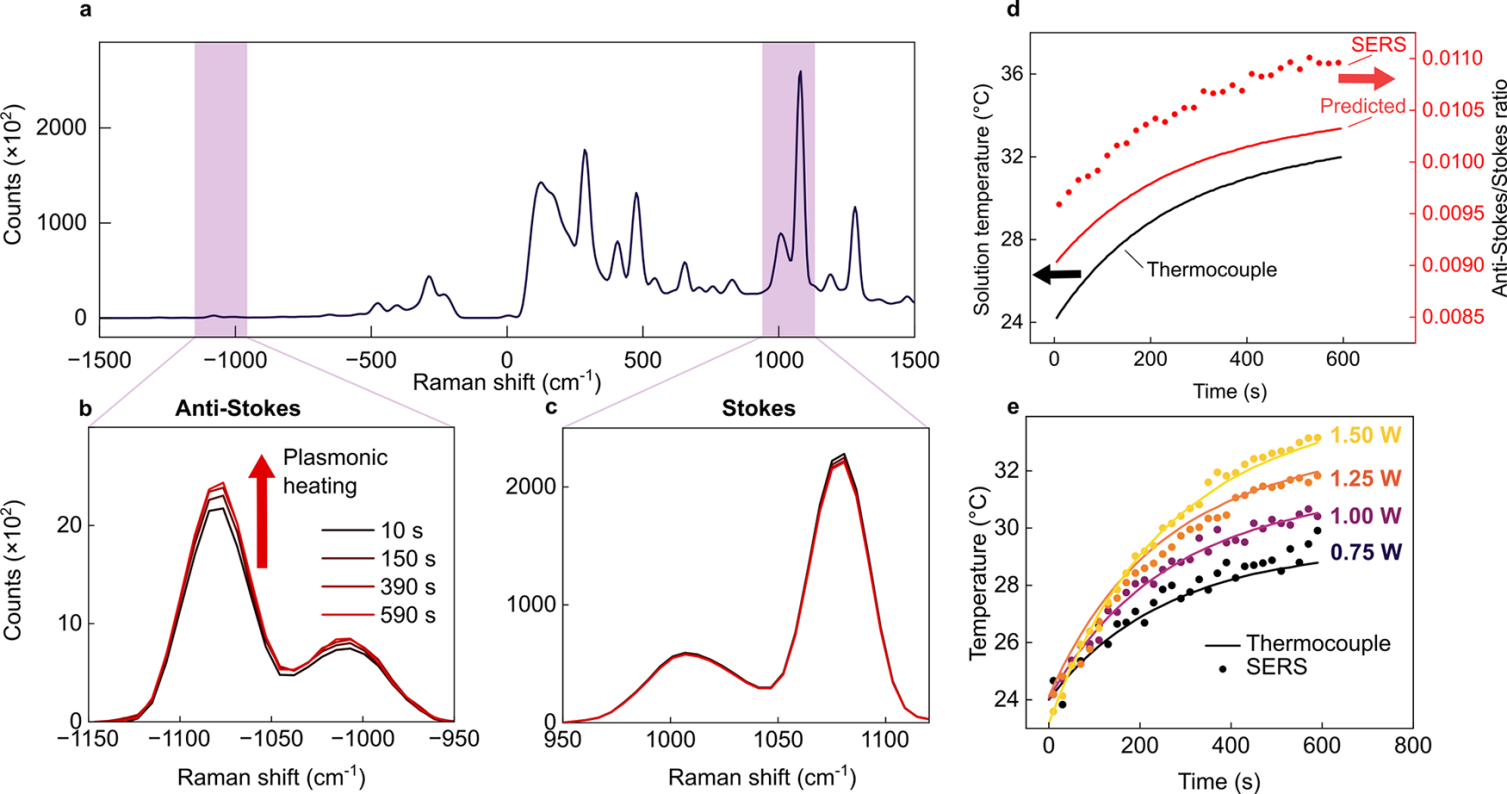
(a) SERS spectrum of BPT-functionalized 40 nm AuNP clusters (808 nm CW laser, 1.25 W, 0.25 s exposure, 20 s accumulation). (b) 1080 cm^–1^ anti-Stokes peak at four time points during 10 min of laser exposure. Anti-Stokes intensity increases as plasmonic heating elevates solution temperature. (c) 1080 cm^–1^ Stokes peak at four time points during 10 min of laser exposure. The intensity remains stable during heating. (d) The solution temperature measured with a thermocouple during heating (black continuous line), the predicted *aS*/*S* ratio (red continuous line) calculated from the thermocouple temperature, and experimental *aS*/*S* ratio measured with SERS (red data points) during 10 min of laser exposure. (e) The temperature measured with SERS and plotted with the thermocouple data during heating with 0.75–1.50 W 808 nm laser (9.25–18.5 W/cm^2^). Data points are the mean temperature increase at each time point, calculated from ν_1080_ and ν_480_ vibrational modes.

To demonstrate the concept of using clusters to heat an environment and measure temperature simultaneously, we collected SERS spectra from a well-mixed colloid of clusters during 10 min of heating with an 808 nm continuous wave (CW) laser (Figure 5a–c). The solution temperature increase was measured with a thermocouple placed away from the laser path, and a total temperature increase of 8 °C was measured. During this time, the intensity of the ν_1080_ anti-Stokes peak increased by ∼15% (Figure 5b), while the Stokes mode remained stable (Figure 5c). To unpick the relationship between solution temperature and the SERS *aS*/*S* ratio, we calculated the predicted ν_1080_*aS*/*S* ratio from 1 (excluding unknown asymmetry factor *A*_*i*_) for the temperatures measured with the thermocouple during heating and plotted these predicted values with the SERS *aS*/*S* ratio measured experimentally (Figure 5d). The SERS and predicted ratios differed in absolute value at all temperatures. Three key factors influence the ratio: asymmetric EM enhancement factors, temperature, and the wavelength-dependent throughput of the optical system (transmission of optics, efficiency of gratings and filters, and quantum efficiency of the detector).

We first set out to determine the relationship between the temperature of BPT molecules in the EM hotspots generating SERS and that of the bulk solution measured with a thermocouple during plasmonic heating. We irradiated the AuNP cluster solution with four laser powers of increasing irradiance (9.25–18.5 W/cm^2^) and measured the resulting ν_1080_ and ν_480_*aS*/*S* ratios and bulk temperature increases (Figure S3–6). The *aS*/*S* ratio of both peaks was constant at any given solution temperature for each applied laser power. This suggests that the temperature in the EM hotspot is approximately equivalent to the surrounding aqueous environment at the instant a SERS photon is scattered and that we are operating within the thermal regime of SERS with no vibrational pumping effects.^16,46^

The temperature of a nanoparticle (δ*T*_*j*_) during plasmonic heating is described by eq 3.^47^ δ*T*_*j*_ is defined by the self-contribution of the nanoparticle being heating (δ*T*_*j*_^self^) and a contribution from the surrounding nanoparticles (δ*T*_*j*_^Ext^).
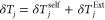
3If δ*T*_*j*_^self^ is dominant, the temperature increase is confined to the immediate vicinity of the nanoparticle. If δ*T*_*j*_^Ext^ is dominant, then thermal collective effects homogenize temperature at the macroscale despite the nanoscale of the plasmonic heaters. Whether nanoparticles are in a temperature confinement or homogenization regime is determined using the dimensionless parameter ξ_m_ in eq S1.2.1. We calculate ξ_3_ ≪ 1 in our system (Supporting Information S1.2), indicating that it is in a homogenization regime. Therefore, our highly localized SERS measurements from EM hotspots reflect the macroscale temperature in the laser spot, which is equal to that measured with the thermocouple in the well-mixed colloid.

The difference between predicted and experimental *aS*/*S* ratio in Figure 5d is, therefore, the result of the system response function and asymmetric EM enhancement of Stokes and anti-Stokes modes. While there are established methods to calibrate the optical system response function, controlling the precise near-field enhancement of plasmonic particles between batches is exceptionally challenging.

Therefore, to achieve SERS temperature measurements, we correct for the impact of asymmetric EM enhancement on *aS*/*S* ratio by applying a single-point calibration for the known starting temperature of the system (*T*_0_, K) and measure temperature elevation in the EM hotspot from the relative *aS*/*S* ratio increase. Figure S3–7 demonstrates close agreement between the relative increase of the SERS and predicted *aS*/*S* ratio during plasmon-driven heating. Hence, because λ_Cluster_, and therefore EM enhancement, remains unchanged during plasmonic heating (Figure S3–8) and the starting temperature is known, a single-point calibration can ensure temperature elevation is accurately measured with SERS nanothermometry using eq 4 (Supporting Information S1.3)
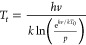
4where *T*_*t*_ is the temperature (K) at time *t* measured with SERS, *h* Planck’s constant, ν the frequency of the vibrational mode, *k* is Boltzmann constant, *T*_0_ is the starting temperature and *p* is the fractional increase in the *aS*/*S* ratio between 0 s and *t*
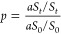
5Using this approach, the increase in temperature at the EM hotspots of the AuNPs clusters can be measured by continuously collecting SERS spectra during a period of plasmonic heating. For our single-point calibration, we measured the solution starting temperature with a thermocouple and estimated the *aS*_0_/*S*_0_ ratio at *t* = 0 s of plasmonic heating by performing linear regression on the first 100 s of spectra (Figure S3–9). The accuracy of our SERS thermometry was confirmed by comparing the temperature increase measured with SERS thermometry to the temperature increase measured simultaneously with a thermocouple in the well-mixed solution (Figure 5e), which yielded a mean RMSE of ±0.3 °C across the four measurements. Higher laser powers lead to smaller standard deviations in the SERS temperature measurement as the intensity and signal-to-noise ratio of the anti-Stokes signal improved. This demonstrates that plasmonic nanoclusters can be used as both nanoheaters and accurate optical thermometers using a single light source. When applied in a biological context to measure plasmonic heating in a tumor, single-point calibration would be performed using physiological temperature (∼37 °C) at *t* = 0 s of laser exposure, enabling accurate noninvasive temperature measurements using SERS only.

In this work, SERS nanothermometry was performed in a well-mixed solution, ensuring uniform heat distribution within the sample. The envisioned application of this technology is in biological matrices. Tumors preferentially accumulate circulating nanoparticles via enhanced permittivity and retention (EPR) and/or active transport and retention (ATR).^48−50^ Hence, temperature gradients are expected to form between regions of high nanoparticle concentration (the tumor) and areas of low nanoparticle concentrations (healthy tissue). Under these conditions, we anticipate that dual nanothermometry and plasmonic heating capabilities will unlock spatially localized temperature measurements in the cellular microenvironment around nanoparticles inaccessible by current techniques. In clinical trials, PTT is typically applied over many minutes.^1^ Hence, our approach could be used to measure AuNP temperature increase above physiological temperature during therapy. Combining this approach with emerging NIR techniques will enable a highly accurate, noninvasive, and spatially localized means of monitoring and directing photothermal therapy.^17^

## Conclusions

We have shown that colloidally stable AuNP assemblies are promising nanostructures for simultaneous photothermal therapy and SERS temperature measurements, with potential future applications in cancer treatment. AuNP building block size in the nanoclusters was tuned to ensure maximum resonance between the NIR laser and chain modes, allowing clusters to act as efficient nanoheaters while generating strong SERS enhancement in the anti-Stokes region. We correlated UV–vis spectra, DLS, and SERS measurements to demonstrate the relationship between the peak plasmon mode, relative cluster size, and EM enhancement of Stokes and the anti-Stoke modes, setting out the thermal stability requirements for subsequent plasmonic heating and anti-Stokes thermometry experiments. Finally, we demonstrated highly accurate anti-Stokes temperature measurements from surface-bound molecules during plasmonic heating. These findings set out a paradigm for the in situ monitoring of plasmonic nanoparticle temperature that could, in the future, be applied to photothermal therapy to guide safe and consistent temperature elevations.

## Materials and Methods

### Nanocluster Assembly

Citrate-stabilized gold nanoparticles (AuNPs) of 30–80 nm diameter were purchased from BBI Solutions, and 14 nm AuNPs were synthesized in-house via a modified Turkevich method.^51,52^ All purchased AuNPs were used at the concentrations received unless stated. The size of these AuNPs was confirmed by transmission electron microscopy (TEM) and dynamic light scattering (DLS) (Table S1–1). AuNP assembly was induced with cucurbit[7]uril (CB[7]) macrocycles^29^ and the kinetic arrest^53^ was performed by adding poly(ethylene glycol) methyl ether thiol (PEG-SH) (Sigma-Aldrich, MW: 6 kDa).^28^ Experimental conditions for aggregation and kinetic arrest are presented in Table S1–2. The nanoclusters were then functionalized by adding biphenyl thiol (BPT) (1 mM dissolved in 0.1 M NaOH) to a final concentration of 1 μM. The UV–vis spectra of each colloid during this sequence is presented in Figure S1–1. AuNP concentration influenced cluster size (Figure S1–2), as well as the time between CB[7] addition and PEG-SH-mediated kinetic arrest (Figure S2–8). Increasing BPT concentrations did not increase the size of the clusters, suggesting that the number of AuNP within a single cluster does not undergo significant changes. However, a BPT concentration of 1 μM was chosen because clusters become unstable at higher BPT concentrations and gradually sediment over time (Figure S1–3).

### Photothermal Properties of Clusters

Nanocluster solutions were diluted to ε_808_ ≈ 0.1 in Milli-Q water for photothermal conversion efficiency (η) and photothermal mass conversion efficiency experiments (η_m_). Two milliliters of the nanocluster solution was placed in a quartz cuvette; mixing was performed with a stir bar designed for rapid horizontal and vertical mixing in cuvettes (Sigma-Aldrich, Z363545), and the solution temperature was monitored with a K-type thermocouple and Pico TC-08 Thermocouple Data Logger. Samples were equilibrated to room temperature before heating. Three independent repeats were performed to calculate η and η_m_ for clusters of each AuNP size. η was calculated using eq 6.^34,35,37^ Each cluster solution was heated with an 808 nm laser (1 W) for 30–40 min and cooled to ambient temperature with the laser switched off.
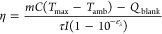
6*m* and *C* are the mass (2 g) and heat capacity (∼4200 J/(kg°C)) of the nanoparticle solution, respectively. *T*_max_ is the temperature at which the solution reaches equilibrium, *T*_amb_ is the ambient temperature, *Q*_blank_ (J) is the energy input by the laser into a blank quartz cuvette filled with 2 mL of water and *I* is the power of the laser beam after attenuation by a cuvette containing Milli-Q water to ensure that only power attenuated by the nanoparticle solution is included in the calculation. ε_λ_ is the extinction of the solution at 808 nm (ε_808_). τ is the time constant calculated with eq 7 using the cooling data after the laser was switched off. In eq 7, *T* is the temperature of the solution and *t* is the time since the laser was turned off.
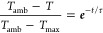
7The photothermal mass conversion efficiency (η_m_) is the heating efficiency of nanoparticles as a function of mass and was calculated for each cluster solution using eq 8.^37^
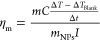
8where *m* and *C* are the mass and heat capacity of the nanoparticle solution. Δ*T* is the temperature increase in the first 60 s after the laser is turned on, Δ*T*_Blank_ is the heating that occurs in a blank cuvette with 2 mL of Milli-Q water and Δ*t* is the duration of heating (1 min). During the first minute of heating, thermal equilibrium with the ambient surroundings is minimal and is approximated to zero in our calculations of η_m_. *I* is the laser power after attenuation by a blank cuvette filled with water and was set to 0.5 W. *m*_NPs_ is the mass of nanoclusters in the path length of the laser (beam area = 0.081 cm^2^ and length = 1 cm). The mass concentration of gold for each sample (mg/mL) was calculated from the UV–vis spectrum of the monodisperse AuNP colloids before aggregation using the method outlined by Haiss et al.^54^ Colloids were diluted in Milli-Q water to ε_808_ ≈ 0.1 for all η_m_ experiments.

### Plasmonic Simulations

Plasmonic coupling along chains of 1–10 AuNPs of 14–80 nm diameter was simulated with MNPBEM, a MATLAB toolbox for simulating metallic nanoparticles using a boundary element method approach.^55^ AuNPs were separated by 0.9 nm and modeled in water.^27,29^

### SERS Comparison of Nanoclusters

Nanocluster solutions were diluted to ε_808_ ≈ 0.25 in Milli-Q water to compare AuNP building block sizes to ensure similar laser attenuation between samples. SERS measurements were performed at 90° to the beam path, and optics are described in the Supporting Information (Section S1.1). The relationship between the peak cluster LSPR (λ_Cluster_) wavelength and relative SERS enhancement in the Stokes and anti-Stokes region was explored by fabricating clusters from 40 nm AuNPs with λ_Cluster_ between ∼690 and ∼810 nm. This was achieved by modulating the concentration of CB[7] added and the duration of assembly before kinetic arrest with PEG and functionalization with biphenyl-4-thiol (BPT). All spectra were acquired with power set to 1 W after attenuation from a blank cuvette and 40 nm AuNP cluster samples diluted to ε_808_ ≈ 0.1. Dynamic light scattering (DLS) measurements were performed with a Malvern Zetasizer Ultra.

### SERS Nanothermometry

Nanoclusters were fabricated with 40 nm AuNPs as previously described and heated for 30 min in a 50 °C water bath to ensure complete assembly of clusters and stable λ_Cluster_ during subsequent heating. The clusters were then diluted to ε_808_ ≈ 0.25 with Milli-Q water. The solution was equilibrated to ambient temperature before heating with 0.75, 1.00, 1.25, and 1.50 W of laser power for 10 min while thoroughly mixing. Spectra were continuously collected with 0.25 s of exposure during heating, and temperature was measured simultaneously with a K-type thermocouple.

### Spectral Processing

The spectra were analyzed with MATLAB. First, they were summed into 20 s acquisition times, followed by linear baseline subtraction of individual Stokes and anti-Stokes peaks at ±480 cm^–1^ and ±1080 cm^–1^. Each peak was fitted with a Gaussian curve to extract the peak intensity.

## Supporting Information Available

CB[7] concentrations used for each AuNP size; optics description; derivation of eq 4; heating and cooling data used to calculate conversion efficiencies; plasmonic modeling of AuNP clusters; SERS spectra from each cluster size; theoretical temperature sensitivity of anti-Stokes modes; cluster plasmon mode wavelength (λ_cluster_) vs ν_480_*aS*/*S* ratio; UV–vis spectra of clusters before and after heating; *aS*/*S* ratio plotted against solution temperature for ν_480_ and ν_1080_ (PDF)
