# Supplementary material for: An All-in-One Nanoheater and Optical Thermometer Fabricated from Fractal Nanoparticle Assemblies
Source: ACS Nano. 2025 Apr 4;19(14):13779–89. doi: 10.1021/acsnano.4c16452 (PMC12005048; doi:10.1021/acsnano.4c16452)
Supplement: Supplementary file 1 — nn4c16452_si_001.pdf [file nn4c16452_si_001.pdf]

An all-in-one nanoheater and optical thermometer fabricated from fractal nanoparticle assemblies

William H. Skinner[1], Renata L. Sala[2], Kamil Sokolowski[2], Ioana Blein-Dezayes[1], Natalie S. Potter[2], Sara Mosca[3], Benjamin Gardner[1], Jeremy J. Baumberg[4], Pavel Matousek[3], Oren A. Scherman[2], Nick Stone[1].*

[1] Department of Physics and Astronomy, University of Exeter, Exeter EX4 4QL, UK.

[2] Melville Laboratory for Polymer Synthesis, Yusuf Hamied Department of Chemistry, University of Cambridge, Lensfield Road, Cambridge CB2 1EW, UK.

[3] Central Laser Facility, Research Complex at Harwell, STFC Rutherford Appleton Laboratory, Oxford OX11 0QX, UK.

[4] Nanophotonics Centre, Cavendish Laboratory, University of Cambridge, Cambridge CB3 0HE, UK.

TABLE OF CONTENTS

Section S1: Methods

Table S1-1. Gold nanoparticle sizing data.

Table S1-2. Volume of CB[7] solution (0.5 mM) and PEG-SH solution (50 µM, 6 KDa) used to cluster and kinetically arrest 2.5 ml of different AuNPs sizes.

Figure S1-1. UV-Vis spectra of cluster fabrication.

Figure S1-2. AuNP starting concentration effect on cluster size.

Figure S1-3. Studies on the influence of BPT concentration on cluster size and SERS

Section S1.1. Optics description

Section S1.2. Collective heating of nanoparticles

Section S1.3. Derivation of equation for SERS temperature measurements

Section S2: Optical and photothermal properties of nanoparticle clusters

Figure S2-1. SERS spectra of 40 nm AuNP clusters before and after adding BPT.

Figure S2-2. Biocompatibility of clusters.

Figure S2-3. Representative heating and cooling data (black**)** and associated plots used to calculate the time constant (τ_s) (red)

Figure S2-4. The complete data set used to calculate photothermal conversion efficiency (ƞ) for each cluster type.

Figure S2-5. Hydrodynamic diameter of AuNP clusters from each AuNP building block size.

Figure S2-6. Heating data used to calculate photothermal mass conversion efficiency (η_m) for clusters fabricated from AuNPs building blocks of 14-80 nm.

Figure S2-7. Plasmonic modeling of AuNP clusters.

Figure S2-8. CB[7]-mediated assembly of AuNPs without kinetic arrest.

Section S3: SERS characterization and temperature sensing

Figure S3-1. SERS spectra of clusters made from each AuNP size.

Figure S3-2. Theoretical relative change in aS/S ratio of ν_{480} and ν_{1080} modes

Figure S3-3. Effect of cluster plasmon mode wavelength ($\lambda_{cluster}$) on ν_{480} aS/S ratio.

Figure S3-4. Effect of cluster plasmon mode wavelength ($\lambda_{cluster}$) on photothermal properties.

Figure S3-5. UV-Vis spectrum of 40 nm AuNPs clusters following multiple 10-minute heating cycles in a 50 °C water bath.

Figure S3-6. aS/S ratio of (a) v_{480} and (b) v_{1080} plotted against solution temperature for 4 laser powers.

Figure S3-7. (a) v_{480} and (b) v_{1080} relative aS/S ratio increase during 600 seconds of plasmonic heating in solution.

Figure S3-8. UV-Vis spectrum of 40 nm AuNPs clusters before and after 4 rounds of plasmonic heating with increasing laser powers (0.75, 1, 1.25, 1.50 W).

Figure S3-9. Calculating the aS/S ratio at t = 0 seconds of laser exposure.

Section S1: Methods

Table S1-1. Gold nanoparticle sizing data

Nanoparticle designation (nm)	Hydrodynamic diameter (±SD, nm)	TEM diameter (±SD, nm)
14	15.9 ± 0.1	12.6 ± 0.8
30	31.9 ± 3.6	29.1 ± 2.6
40	40.4 ± 1.5	40.6 ± 3.7
60	57 ± 2.9	60.2 ± 5.8
80	88.9 ± 1.8	82.2 ± 8.3

Table S1-2. Volume of CB[7] solution (0.5 mM) and PEG-SH solution (50 µM, 6 KDa) used to cluster 2.5 ml of different AuNPs sizes.

AuNPs size (nm)	Volume of CB[7] (µl)	Volume of PEG-SH (µl)	Time before kinetic arrest (minutes)
14	7	42	2
30	10	5	3
40	10	4.5	3
60	20	2.5	2
80	40	2	2

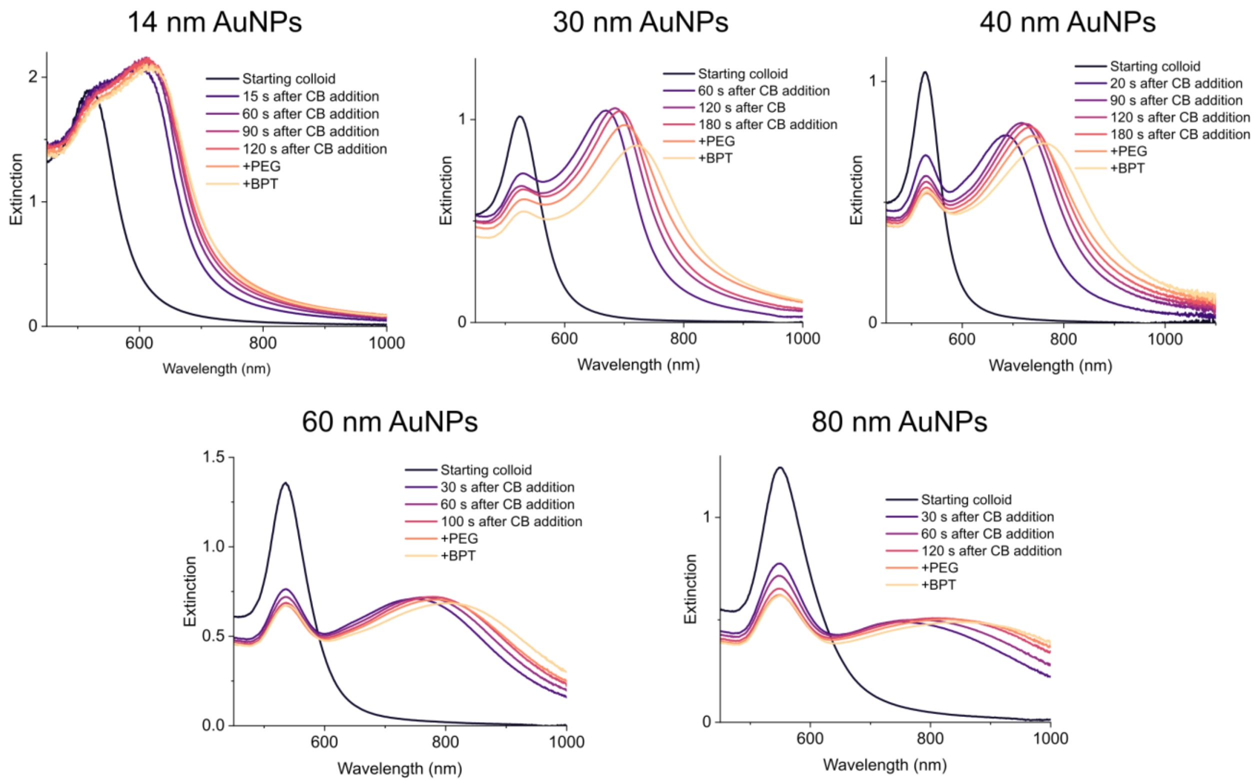

Figure S1-1. UV-vis spectra of each AuNP size during CB[7]-induced assembly, subsequent PEG-SH kinetic arrest and BPT functionalization.

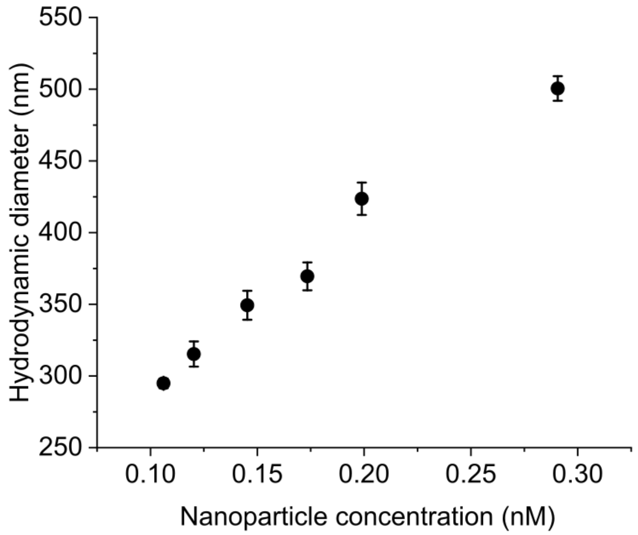

Figure S1-2. 40 nm AuNP cluster size compared to the concentration of starting AuNP colloid. The same volume of CB[7] was used for each data point (Table S1-2), and the kinetic arrest was performed at the same time point for each sample. The hydrodynamic diameter was measured with DLS. AuNP were concentrated via centrifugation before CB[7] addition. The hydrodynamic diameter in these data is slightly larger than in Figure 4, S1-3 and S2-5. The data were collected

by a different operator working in a different laboratory than those presented in Figure 4, S1-3 and S2-5. We ascribe the larger hydrodynamic diameter in these results to slight differences in the rate of nanoparticle assembly between AuNP batches and operator variability.

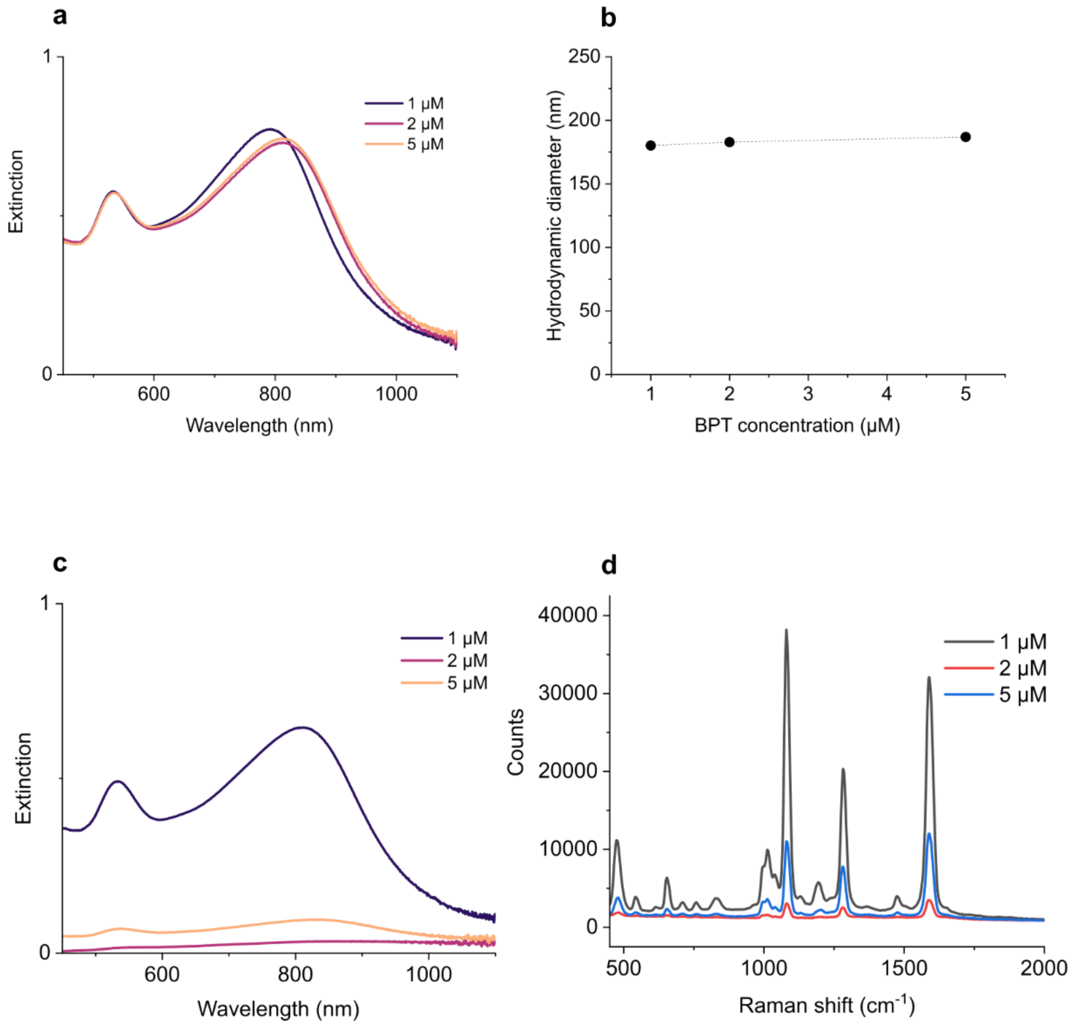

Figure S1-3. Studies on the influence of BPT concentration on optical properties and colloidal stability of 40 nm AuNP clusters. (a) UV-Vis spectra of clusters 120 minutes after adding 1, 2, and 5 μM of BPT. (b) The hydrodynamic diameter of clusters 120 minutes after fabrication with each BPT concentration. (c) UV-Vis spectra of clusters 24 hours after fabrication. (d) SERS spectra of clusters 24 hours after fabrication.

Section S1.1 Optics description

An 808 nm solid-state laser (Innovative Photonic Solutions, Monmouth Junction, NJ, USA) was coupled with a 550 μm core fiber (0.22 NA, Thorlabs, M37L02) and collimated with a fiber collimator (Thorlabs, CVH100-COL). The collimated beam then passed through a single 808 nm laser clean-up filter (LL01-808-25, Semrock, Rochester, New York, USA). The beam spot area at the sample was 8.1 mm^2. Raman spectra were collected at 90° to the illumination path, filtered with an 808 nm notch filter (Kaiser Optical Systems, Ann Arbor, Michigan, USA), and focused

onto a collection bundle (Ceramoptec) with a circular array of fibers at the collection end and a linear array of fibers. The bundle was coupled to a Holospec 1.8i (Kaiser Optical Systems) spectrometer containing an additional 808 nm notch filter (Kaiser Optical Systems), a 167 µm slit and Kaiser VPH Grating HFG 927. The spectrometer was coupled to a deep depletion CCD detector (Andor BR-DD iDus 420) cooled to -70°C to record the Raman spectra.

Section S1.2 Collective heating of nanoparticles

The heating regime of a nanoparticle dispersion can be determined using Equation S1.2.1.[1] Where p is the neighboring nanoparticle distance, a is the nanoparticle radius, N is the number of nanoparticles in the system, and m is the system's dimensionality. For a dispersion of nanoparticles, m=3.

$$\zeta_m = \frac{p}{aN^{(m-1)/m}} \qquad \text{(S1.2.1)}$$

We measured the concentration of 40 nm AuNP clusters with a Nanosight (LM10, Malvern Panalytical) (3.16×10^{9} clusters/ml), the hydrodynamic radius with DLS, and N was calculated for the number of clusters in the laser path volume. The values in Table S1-3 were used to calculate ζ_3 which was found to be <1, demonstrating the system is in a temperature homogenization regime.

Table S1-3 Parameter values used to calculate ζ for plasmonic heating of 40 nm AuNP cluster colloids in Figure 5.

Parameter	Value
p	6.8×10^{-6} m
a	90 nm
N	2.5×10^{8}
$\boldsymbol{\zeta_3}$	1.9×10^{-4}

Section S1.3 Derivation of equation for SERS temperature measurements

For Raman spectra collected with a charged coupled device detector (CCD), the ratio of the anti-Stokes to Stokes peaks of a vibrational mode is linked to temperature through Equation 1.3.1, derived from Boltzmann's distribution.

$$aS/S = A_i \frac{(v_i+v_v)^3}{(v_i-v_v)^3} e^{\left(\frac{-hv}{kT}\right)} \qquad \text{(S1.3.1)}$$

where aS/S is the ratio of a specific vibrational mode, A_i is the average asymmetry factor from resonance effects, v_i is the laser frequency, v_v is the frequency of the vibrational mode, k is the Boltzmann constant, h is Plank's constant and T is temperature (K). For PTT, it is important to measure the temperature increase above physiological temperature (i.e. the starting temperature,

T_0), hence we measure the relative increase (p) of aS/S for a specific vibrational mode between time 0 and t:

$$p = \frac{aS_t/S_t}{aS_0/S_0} \qquad \text{(S1.3.2)}$$

substituting (S1.3.1) into (S1.3.2) for each time point:

$$p = \frac{e^{(\frac{-hv}{kT_t})}}{e^{(\frac{-hv}{kT_0})}} \qquad \text{(S1.3.3)}$$

which can be rearranged to (S1.3.4):

$$T_t = \frac{hv}{kln(\frac{e^{\frac{hv}{kT_0}}}{p})} \qquad \text{(S1.3.4)}$$

which allows T_t to be calculated from a relative increase in aS/S from a known starting temperature.

Section S2: Optical and photothermal properties of nanoparticle clusters

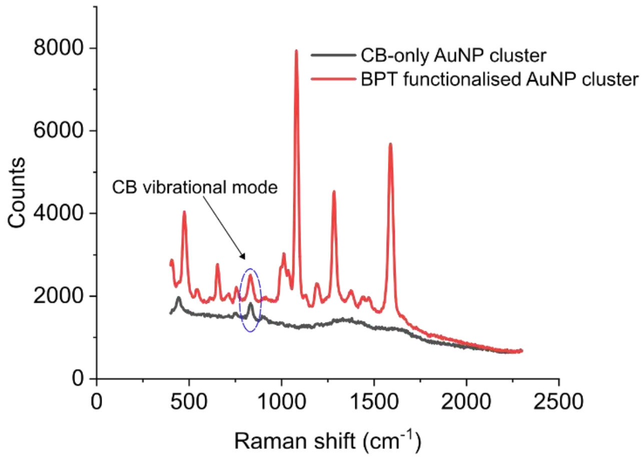

Figure S2-1. SERS spectra of 40 nm AuNP clusters before and after adding BPT Raman reporter molecule. Both clusters were stabilized with PEG, as described in the methods section. The stronger SERS generated by BPT compared to CB[7] reflects its larger Raman cross-section.

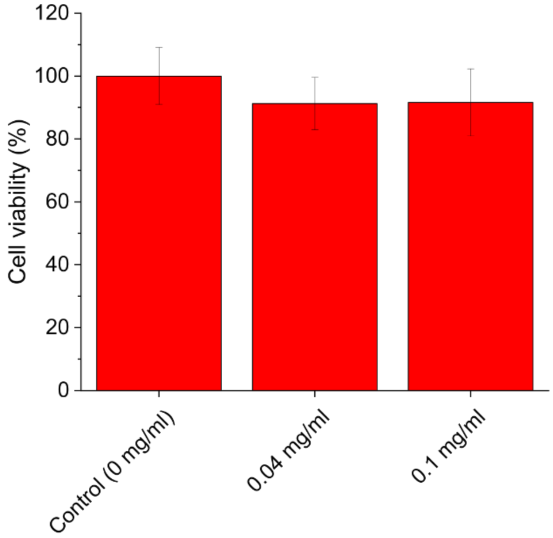

Figure S2-2. WST-1 cell viability assay after 24 hours of incubation with nanoclusters concentrations used in literature to test photothermal heating of nanoparticles in vitro[1]. Murine breast cancer cells (4T1 cell line) were plated in a 96-well plate. After 24-hour incubation, the cell culture medium (DMEM, 10% fetal bovine serum, 1% Pen-Strep) was replaced with 100 µl of medium containing 0, 0.04, and 0.1 mg/ml of 40 nm AuNP clusters. Clusters were concentrated by centrifugation at 750 RCF for 10 minutes in LoBind® centrifuge tubes (Eppendorf) and resuspended in cell culture medium. Cells were incubated for a further 24 hours with the clusters and control before washing with phosphate-buffered saline and performing the WST-1 assay (CELLPRO-RO, Roche). The assay was read on the INFINITE F50 (Tecan Life Sciences) plate reader. n=3 repeats were performed for each condition.

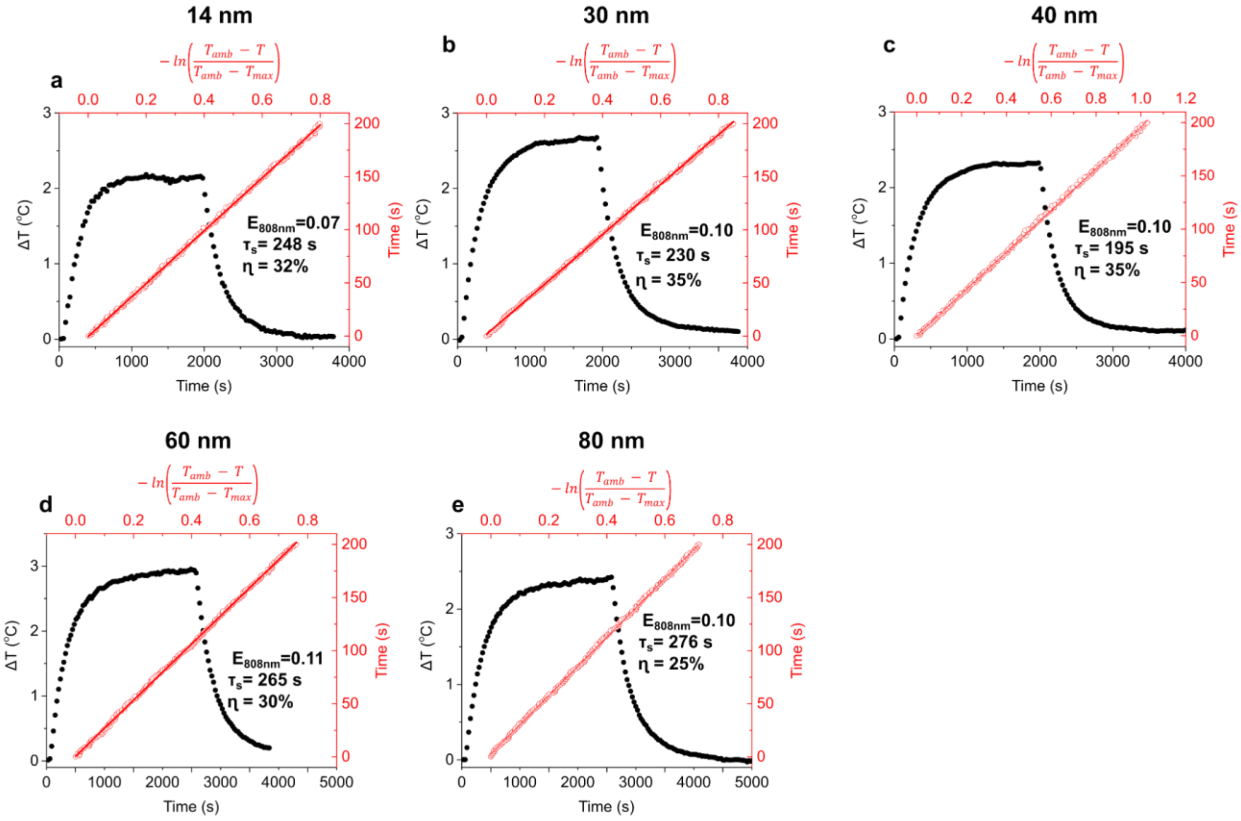

Figure S2-3. Representative heating and cooling data (black) and associated plots used to calculate the time constant (τ_s) from cooling data (red). Samples were made from each AuNPs building block size: (a) 14 nm, (b) 30 nm, (c) 40 nm, (d) 60 nm and (e) 80 nm. The photothermal conversion efficiency (η) of each cluster type was calculated using Equation 6. Clusters were diluted with Milli-Q water to an extinction of 0.07-0.12 at the laser wavelength (808 nm, 1 W) and heating was performed in a well-mixed 2 mL volume.

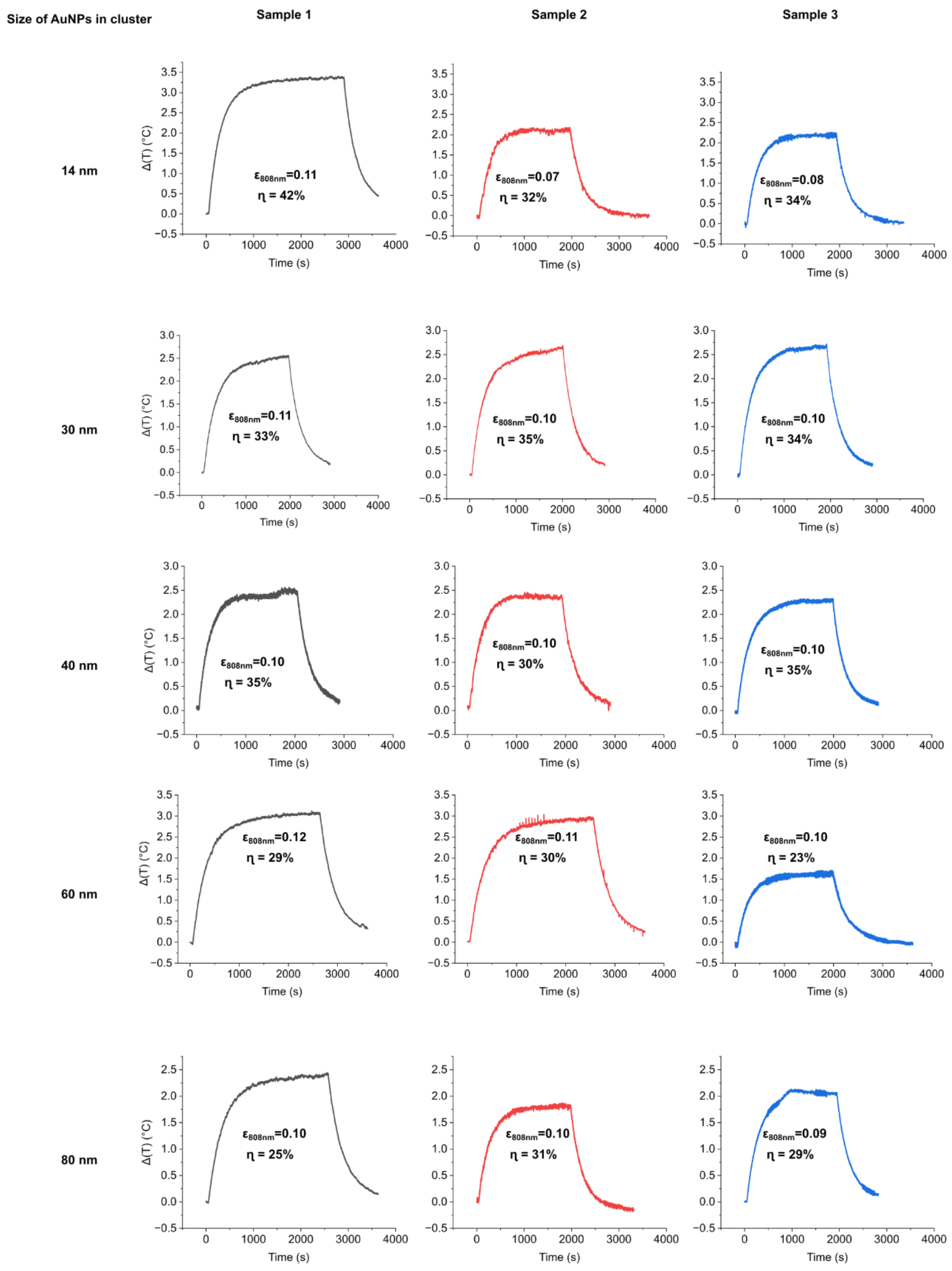

Figure S2-4. The complete data set used to calculate photothermal conversion efficiency (η) for each cluster type. Three cluster samples were made from each AuNP size. Each sample was diluted to an extinction between 0.07-0.12 at 808 nm (ε_{808nm}) in a 2 ml volume and exposed to an 808 nm

laser (1 W). Samples were mixed thoroughly with a stir bar during heating. η was calculated using Equation 6.

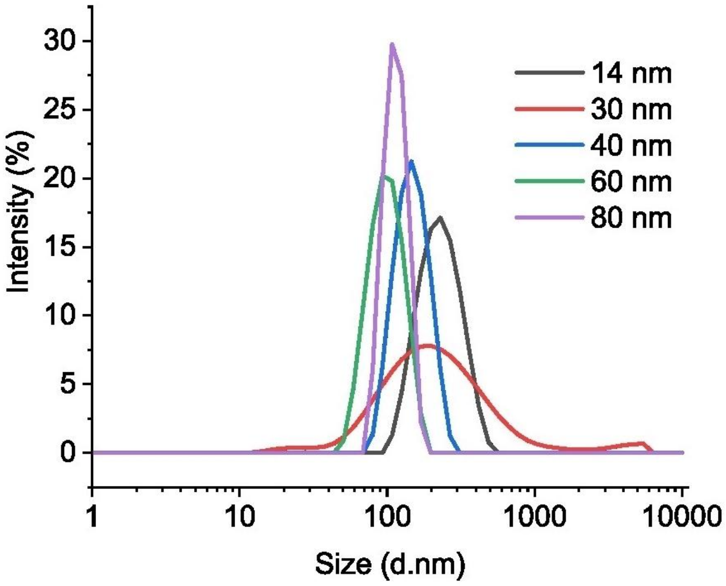

Figure S2-5. DLS sizing of AuNP clusters tuned to wavelengths presented in Figure 2a. Generally, there is a trend towards smaller clusters when larger base AuNPs are used. This is consistent with the assembly times in Table S1-2 and the modeling in Figure S2-7, which shows that longer AuNP chains are required to generate modes close to the 808 nm laser when small AuNPs are used. Clusters fabricated from 14 nm AuNP had the largest hydrodynamic diameter, most likely because of their faster assembly kinetics (Figure S2-8). The hydrodynamic diameter measured with DLS can be approximated as the diameter of a sphere of volume equal to the fractal nanoclusters.[2] The effective volume (V) of clusters scales linearly with the number of nanoparticles in the cluster. However, the effective diameter scales as $V^{1/3}$. Therefore, DLS measurements are likely to significantly underestimate the longest dimensions of fractal nanoparticle clusters.

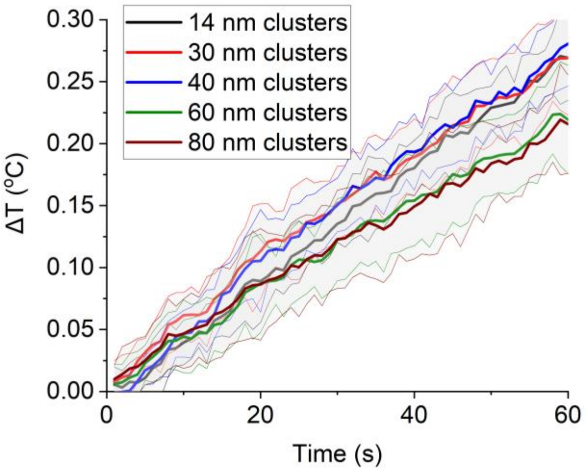

Figure S2-6. Heating data used to calculate photothermal mass conversion efficiency (η_m) for clusters fabricated from AuNPs building blocks of 14-80 nm. All clusters were diluted to an extinction of ~0.1 at the laser wavelength (808 nm) and heating was performed in a well-mixed 2 mL volume using 0.5 W of laser power.

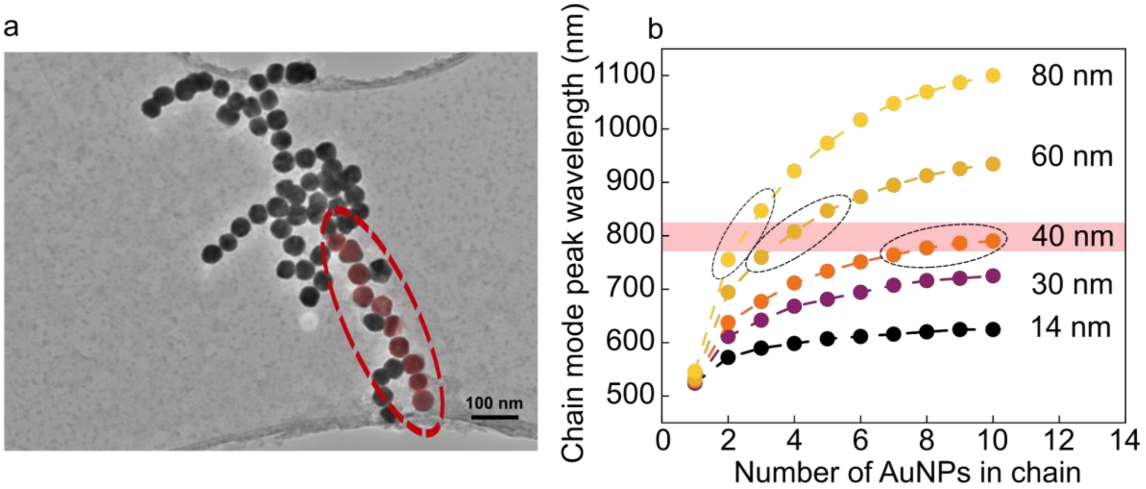

Figure S2-7. Plasmonic modelling of AuNP clusters. (a) TEM image of 40 nm clusters. Fractal clusters are made up of chains of AuNPs separated by CB[7] (0.9 nm), an embed chain substructure is highlighted in red within the larger cluster. (b) Simulation of plasmon mode wavelength of idealized straight chains of 1-10 AuNPs of sizes 14-80 nm with separation of 0.9 nm (diameter of CB[7]). The red-shaded area highlights the laser wavelength (808 nm). Nanoparticle chain lengths in resonance and close to resonance are circled with a dotted line.

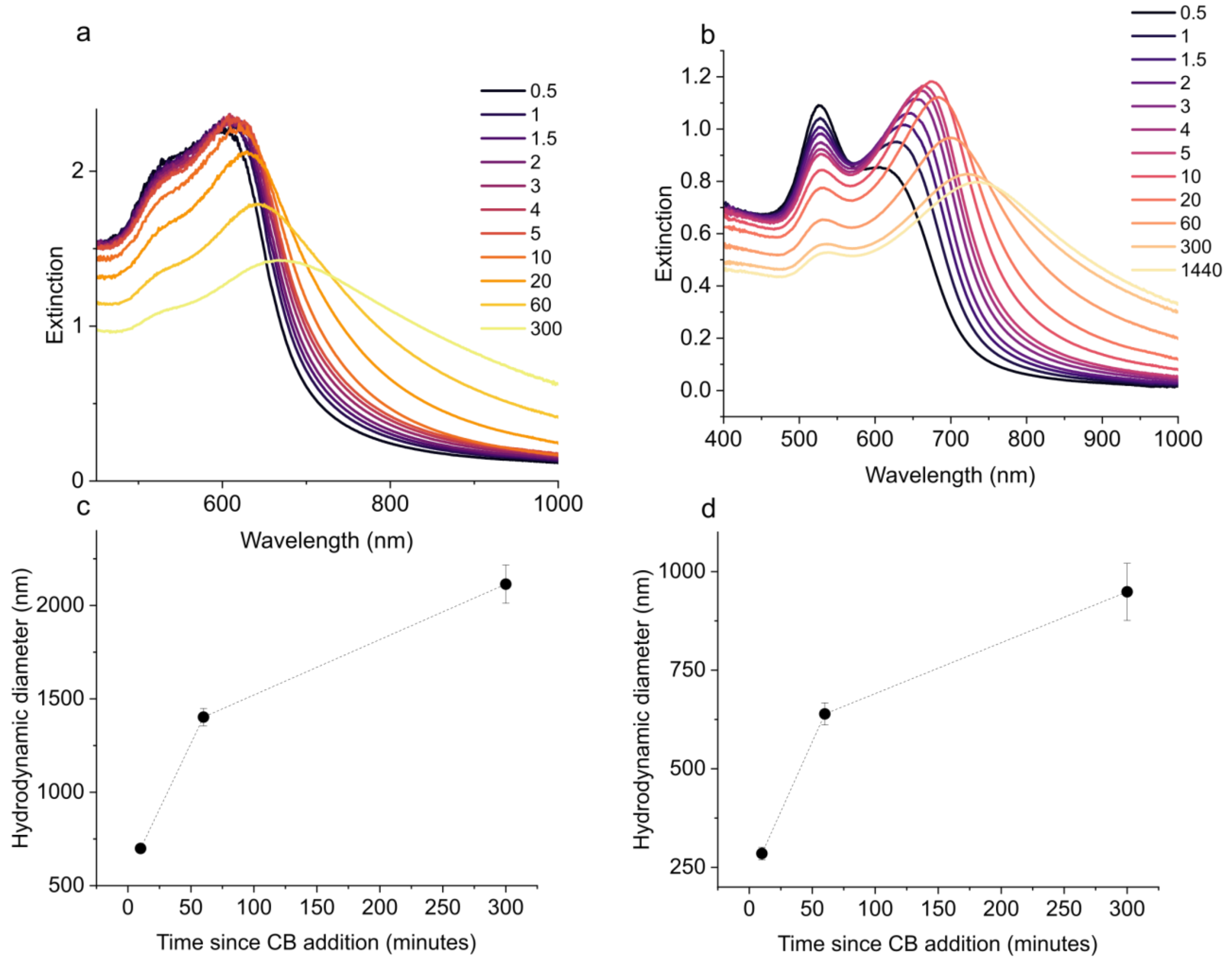

Figure S2-8. CB[7]-mediated assembly of 14 nm and 30 nm AuNPs without kinetic arrest. (a) Extinction spectra of 14 nm AuNP at 0.5-300 minutes after CB[7] was added to the colloid. (b) Extinction spectra of 30 nm AuNP at 0.5-1440 minutes after CB[7] was added to the colloid. (c) Hydrodynamic diameter (measured with DLS) of 14 nm clusters at 10, 60 and 300 minutes after CB[7] addition. (d) Hydrodynamic diameter of 30 nm clusters at 10, 60 and 300 minutes after CB[7] addition. Nanoparticle clusters continue to grow if they are not kinetically arrested with PEG-SH. Growth of larger clusters ~1 μm in diameter produces only slightly larger LSPR redshifts. This is consistent with modeling in S2-7, which shows diminishing redshifts as AuNP are added to longer chains. The majority of the redshift occurs in the first < 10 minutes during short chain growth in the clusters. For 30 nm AuNPs, a ~Δ160 nm shift occurs in the first 10 minutes, and the subsequent 300 minutes produce a redshift of ~Δ40 nm. During this time, the hydrodynamic diameter more than triples.

Section S3: SERS characterization and temperature sensing

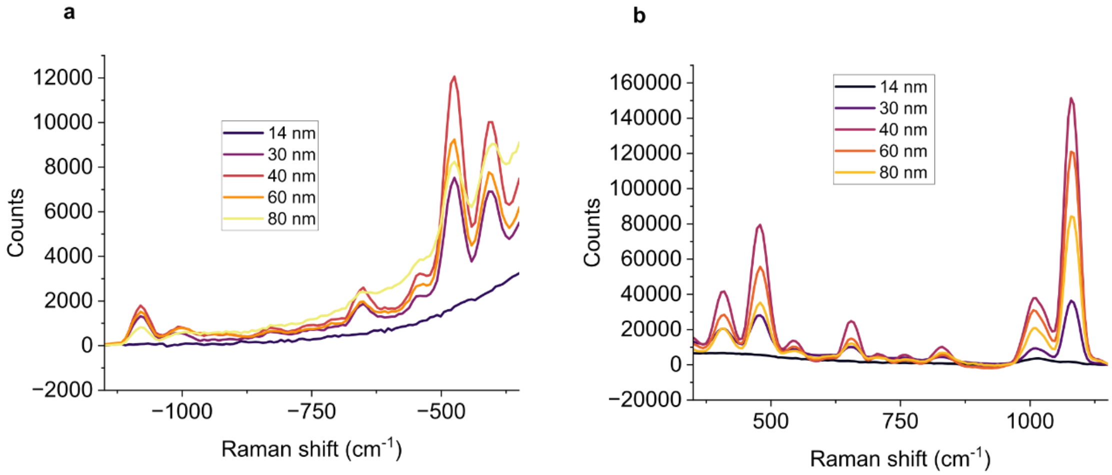

Figure S3-1. Complete SERS spectra of clusters made from each AuNP size. (a) anti-Stokes SERS spectrum of each cluster type without baseline subtraction. (b) Stokes SERS spectrum of each cluster type without baseline subtraction.

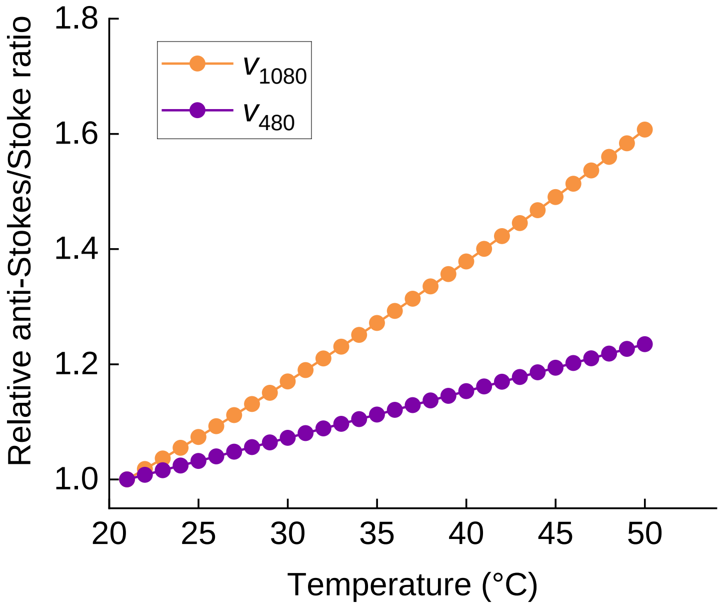

Figure S3-2. Theoretical relative change in aS/S ratio of ν_{480} and ν_{1080} modes from room temperature to body temperature and temperatures required for tumor photothermal therapy (37-50 °C). ν_{1080} has a greater temperature sensitivity than ν_{480}. However, ν_{1080} is much weaker than ν_{480} (Figure 3a). Values plotted calculated from Equation 1 in the absence of the asymmetry factor.

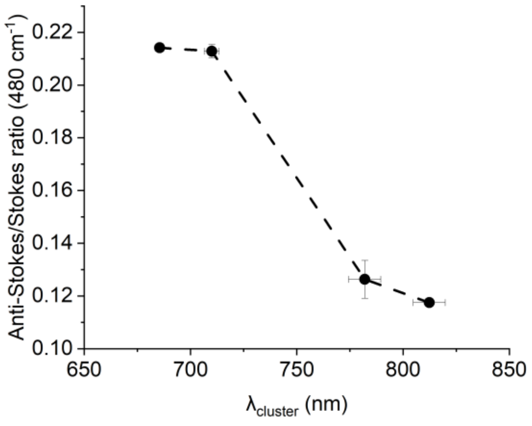

Figure S3-3. Effect of cluster plasmon mode wavelength ($\lambda_{cluster}$) on v_{480} aS/S ratio. Clusters were fabricated from 40 nm AuNPs and $\lambda_{cluster}$ adjusted by lowering CB[7] concentration or increasing CB[7] assembly time before adding PEG and BPT functionalization.

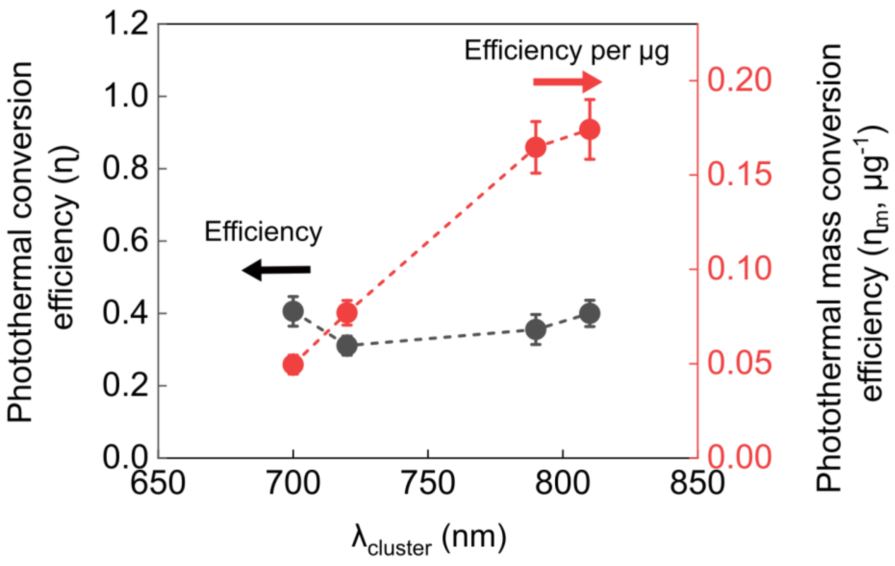

Figure S3-4. The photothermal conversion efficiency (η) and photothermal mass conversion efficiency (η_m) of four 40 nm AuNP clusters with increasingly redshifted plasmon modes and larger sizes. η_m increases as the plasmon mode redshifts because the overlap with the laser line at 808 nm increases. η remains near-constant as the plasmon mode redshifts and cluster size increases, indicating that, over the range measured, the fraction of laser light absorbed in our experiment does not change significantly as cluster size increases and plasmon mode redshifts. These results are consistent with Figure 2 and the accompanying discussion.

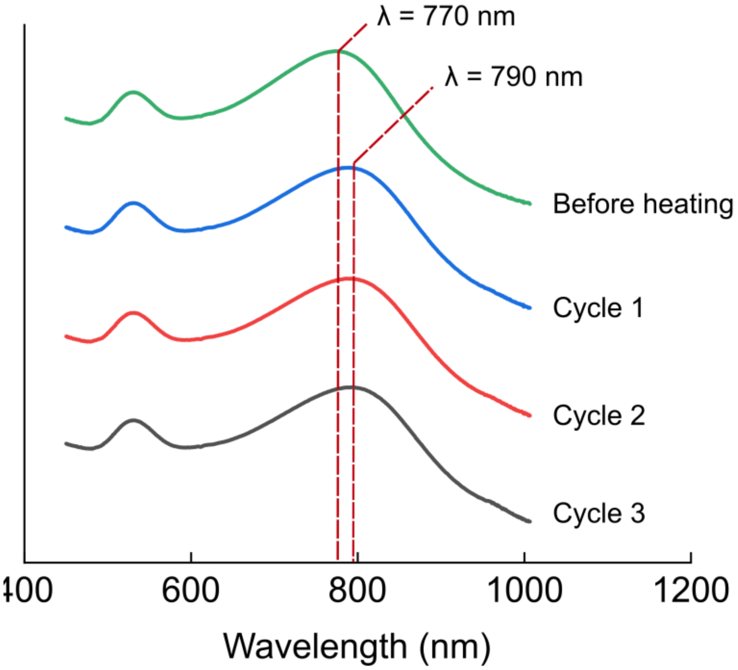

Figure S3-5. UV-Vis spectrum of 40 nm AuNPs clusters following multiple 10-minute heating cycles in a 50 °C water bath.

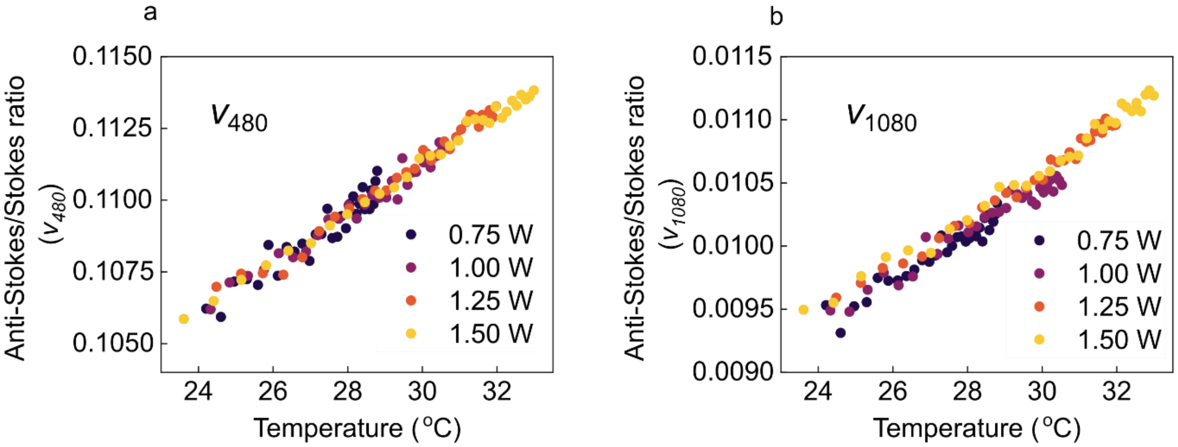

Figure S3-6. aS/S ratio of (a) ν_{480} and (b) ν_{1080} plotted against solution temperature for 4 laser powers. The ratio for each mode is consistent at a given solution temperature when heated with different laser powers. These data indicate the temperature in the EM hotspot when a SERS photon is scattered is essentially equivalent to that of the bulk aqueous environment of the colloid for the power regime explored in this work. Each data point represents the mean ratio aS/S over 20 seconds. Beam area = 0.081 cm^2.

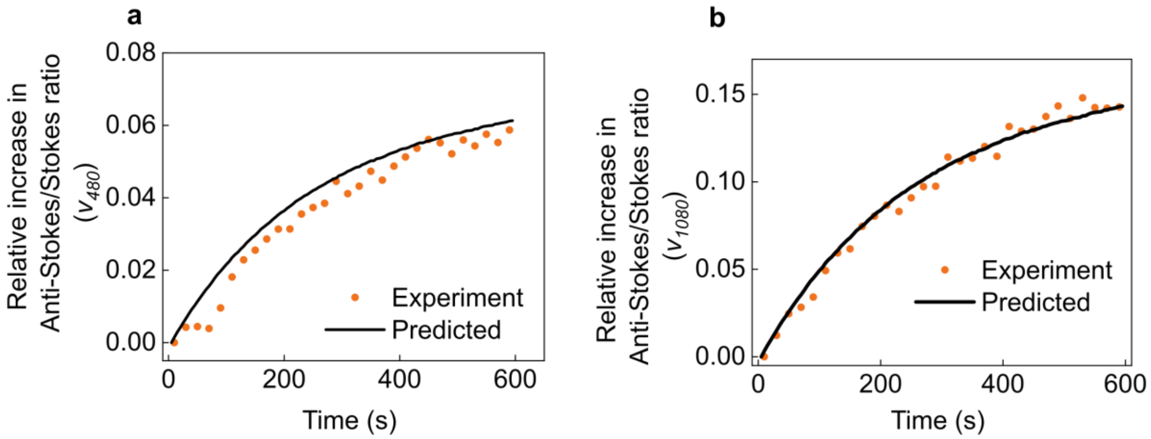

Figure S3-7. (a) v_{480} and (b) v_{1080} relative aS/S ratio increase during 600 seconds of plasmonic heating in solution. 'Predicted' values were calculated from the relative increase of thermocouple temperature (Figure 5d) and 'Experiment' data points were calculated from the relative increase in SERS spectra collected during heating. Laser power = 1.25 W, spectral data accumulated over 20 s.

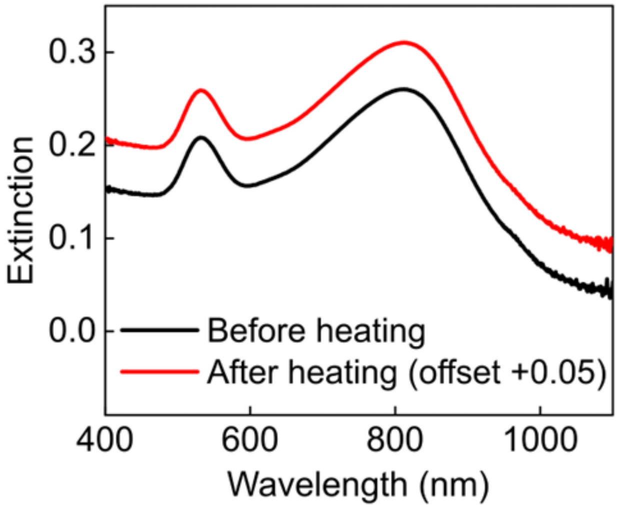

Figure S3-8. UV-Vis spectrum of 40 nm AuNPs clusters before and after 4 rounds of plasmonic heating with increasing laser powers (0.75, 1, 1.25, 1.50 W).

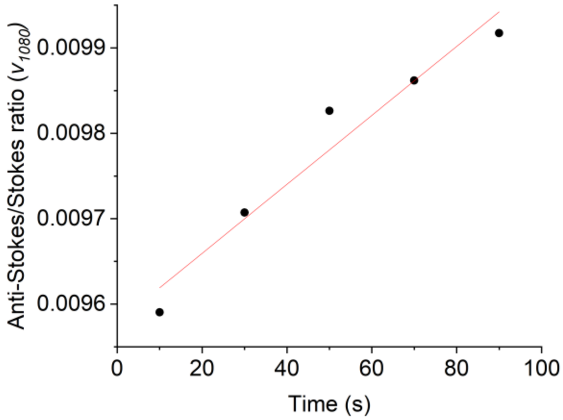

Figure S3-9. Calculating the aS/S ratio at t = 0 seconds of laser exposure. A plot of the aS/S ratio of v_{1080} during the first 100 seconds after the laser is switched on (808 nm, 1.25 W). Linear regression was used to estimate the aS/S ratio at time = 0 seconds i.e. the ratio at the starting temperature before the effects of plasmonic heating. Anti-Stokes/Stokes ratio at 0 seconds = 0.00958.

References

(1) Baffou, G. *Thermoplasmonics: Heating Metal Nanoparticles Using Light*, 1st ed.; Cambridge University Press, 2017. https://doi.org/10.1017/9781108289801.

(2) Pérez-Hernández, M.; del Pino, P.; Mitchell, S. G.; Moros, M.; Stepien, G.; Pelaz, B.; Parak, W. J.; Gálvez, E. M.; Pardo, J.; de la Fuente, J. M. Dissecting the Molecular Mechanism of Apoptosis during Photothermal Therapy Using Gold Nanoprisms. *ACS Nano* **2015**, *9* (1), 52–61. https://doi.org/10.1021/nn505468v.

(3) Lotya, M.; Rakovich, A.; Donegan, J. F.; Coleman, J. N. Measuring the Lateral Size of Liquid-Exfoliated Nanosheets with Dynamic Light Scattering. *Nanotechnology* **2013**, *24* (26), 265703. https://doi.org/10.1088/0957-4484/24/26/265703.
